# FPGA Applied to Latency Reduction for the Tactile Internet

**DOI:** 10.3390/s22207851

**Published:** 2022-10-16

**Authors:** José C. V. S. Junior, Sérgio N. Silva, Matheus F. Torquato, Toktam Mahmoodi, Mischa Dohler, Marcelo A. C. Fernandes

**Affiliations:** 1 Laboratory of Machine Learning and Intelligent Instrumentation, Federal University of Rio Grande do Norte, Natal 59078-970, RN, Brazil; 2 Centre for Telecommunications Research, Department of Engineering, King’s College London, London WC2R 2LS, UK; 3 Department of Computer Engineering and Automation, Federal University of Rio Grande do Norte, Natal 59078-970, RN, Brazil

**Keywords:** tactile internet, latency reduction, haptic devices, FPGA

## Abstract

Tactile internet applications allow robotic devices to be remotely controlled over a communication medium with an unnoticeable time delay. In bilateral communication, the acceptable round trip latency is usually 1 ms up to 10 ms, depending on the application requirements. The communication network is estimated to generate 70% of the total latency, and master and slave devices produce the remaining 30%. Thus, this paper proposes a strategy to reduce 30% of the total latency produced by such devices. The strategy is to use FPGAs to minimize the execution time of device-associated algorithms. With this in mind, this work presents a new hardware reference model for modules that implement nonlinear positioning and force calculations and a tactile system formed by two robotic manipulators. In addition to presenting the implementation details, simulations and experimental tests are performed in order to validate the hardware proposed model. Results associated with the FPGA sampling rate, throughput, latency, and post-synthesis occupancy area are analyzed.

## 1. Introduction

Tactile internet is conceptually defined as the new generation of internet connectivity which will combine very low latency with extremely high availability, reliability and security [1,2]. Another feature that has been pointed out is that this new generation will be centered around applications that use human-machine communications (H2M) alongside devices that are compatible with tactile sensations [3,4,5]. Currently, the IEEE 1918.1 standard [6] defines characteristics of the tactile internet, where both the structure and description of application scenarios and definitions are presented. A tactile internet environment is basically composed of a local device (known as a master) and a remote device (known as a slave), where the master device is responsible for controlling the slave device over the internet through a two-way data communication network [7,8]. Bidirectional communication is needed to simulate the physical laws of action and reaction, where action can be represented as sending operational commands and reaction can be represented as the forces resulting from that action. In tactile internet applications, the desired time delay for device communication is characterized by an ultra-low latency. In bilateral communication, the required round trip latency ranges from 1ms up to 10ms depending on the application requirements [9,10,11,12].

According to [13], it can be noticed that in a tactile internet application, 30% of the total system latency is generated by the master and slave devices. These devices demand high processing speeds as repeated execution of a variety of computationally expensive algorithms and techniques are required. These algorithms involve the use of arithmetic operations and calculations of linear and nonlinear equations that need to be computed at high sampling rates in order to maintain application fidelity. The remaining 70% of the latency is caused by the communication network, which makes them unsuitable for such latency constraints [14]. To address this problem, some research groups have been studying communication networks in the context of tactile internet. The works [15,16,17] shows some types of techniques that can be used to reduce network latency.

Other groups have been studying prediction techniques, where many algorithms have been studied and proposals using artificial intelligence (AI) have proved to be effective [18]. On the other hand, the implementation of complex AI-based prediction methods can further increase the latency of the computer systems present in master and slave devices. Alternatively, new approaches such as using field-programmable gate arrays (FPGAs) can improve the performance of master and slave devices in a tactile system environment. The FPGAs enables the creation of customizable hardware which allow algorithms to be parallelized and optimized at the logical gate level to speed up their operations. Literature results show that computationally expensive algorithms can achieve speedups of up to 1000× over software implementations when custom-implemented in FPGAs [19,20,21,22,23,24,25].

In this context, this paper has, with motivation, a hardware proposed implementation to target reducing the 30% of the total latency related to tactile devices. The project uses FPGA devices to minimize the execution time of algorithms associated with master and slave devices. FPGAs allow the parallelization of algorithms and latency reduction compared to software systems embedded in traditional architectures with general purpose processors and microcontrollers. In an effort to validate the proposed strategy, this paper presents a discrete reference model that can be adjusted for different types of master and slave devices in a tactile internet system. Validation results, throughput, and post-synthesis figures obtained for the proposed hardware implementation using FPGA devices are presented. Comparisons with other works in the literature show that using FPGA can significantly accelerate the processing speed in tactile devices. Thus, this work makes the following contributions:

- A new discrete reference model for a tactile internet system.
- The novel reference architecture for hardware design on FPGA for tactile master and slave devices.
- A new reference architecture for forward and inverse kinematics on FPGA.
- A new strategy to reduce the latency on tactile internet based on FPGA.
- Comparison of performance of the proposed hardware model with other proposals in the literature.

The remainder of this paper is organized as follows: Section 2 presents the related works in the literature; Section 3 introduces a new discrete reference model for a tactile internet system; Section 4 describes the PHANToM Omni robot used with master and slave device; Section 5 presents the simulated tactile internet model; Section 6 gives all detailed description of the reference hardware architectures proposed in this paper; Section 7 presents and analyzes the synthesis results obtained from the described implementation, including a comparison to other works; Section 8 presents the final considerations.

## 2. Related Work

The authors of [26] presented a tactile internet environment that used a glove type device in conjunction with a robotic manipulator. The environment was developed using a general purpose processor, which made the execution of the algorithms sequential. In order to send the data, the tactile glove produced a latency of approximately 4.82ms, and the hardware responsible for performing the inverse kinematics calculations took an interval of 0.95ms. The latency values obtained in this application could be improved by hardware structures that allow algorithms parallelization.

Studies in the literature demonstrate the benefit of using FPGA to accelerate the sample rate for data acquisition from devices associated with haptic systems. The authors of [27] presented an implementation for controlling a 3-DoF (Degree of Freedom) device. The presented technique proposed to increase the device sampling rate using FPGA hardware together with a real-time operating system (RTOS) in order to increase the resolution acquisition of the stiffness sensor. The control technique presented was developed in 32-bit fixed point, and trigonometric functions were implemented using lookup tables.

The work described in [28] presented a control system for one-dimensional haptic devices (1-DoF). The FPGA control implementation used single-precision floating point representation (IEEE std 754) and the algorithms performed all calculations in 50μs. The processing time was satisfactory; however, the data frame size to be sent over the network increased with the size of the DoF. This peculiarity can increase latency for more complex haptics systems with many DoFs. In the same topic of previous works, an implementation for bilateral control of single-dimensional haptic devices (1-DoF) was presented in [29]. A more accurate control techniques based on the sliding mode control (SMC) was implemented in FPGA, and to assist in performing the complex calculations, the CORDIC (COordinate Rotation DIgital Computer) was used. The hardware was designed to locally control two devices, one master and one slave. In the implementation, a 24-bit fixed point was used, of which 9 bits in the integer part and 14 bits for the fractional, and the total execution time of the controllers was of 7.2375μs.

The works [27,28,29] presented a control that depends directly on the encoder reading of the device motors. Usually in commercial models, accessing the device electronics can be tricky requiring some reverse engineering and specific knowledge to make the appropriate encoder connections. On the other hand, some works abstract the data acquisition and work directly with robotics algorithms. These algorithms may require high computational power that can surpass the capabilities of many general-purpose processors (GPPs) that perform the operations sequentially.

Some studies demonstrate the benefit of using FPGA to accelerate robotic manipulation algorithms related to haptic systems. A hardware architecture implemented in FPGA for performing the forward kinematics of 5-DoF robots using floating point arithmetic was described in [30]. In this hardware implementation, all the forward kinematics calculations were performed within 1.24μs which represents 67 clock cycles in a frequency of 54MHz. The equivalent software implementation has a total processing time of 1.61036ms. Overall, the hardware implementation is 1298× faster than the software implementation, which means a considerable acceleration in the forward kinematics processing time.

The authors of the paper [31] presented an FPGA implementation of inverse kinematics, velocity calculation and acceleration of a 3-DoF robot. Three systems were created: the first one did not use any arithmetic co-processor and floating point operations were performed in software; in the second system a floating point co-processor was used which allowed the execution of the four basic mathematical operations in hardware; lastly, the third system also had a custom arithmetic co-processor but in this case it allowed hardware computation of square root. The overall times to perform the calculations were 2324μs, 560μs and 143μs and the total logic elements used from the entire device were 4501 (4%), 5840 (5%) and 7219 (6%), respectively. The work uses hardware–software to implement inverse kinematics, in which critical parts were implemented in FPGAs to accelerate the whole process.

In [32], a hardware able to control a 6-DoF device using 32-bit fixed point representation is presented, where 21 bits were used for the fractional part and 11 bits for the integer part. In that work, a CORDIC implementation was used to assist in performing the trigonometric calculations. The total time spent to compute the forward kinematics was 3μs and for the inverse kinematics the time was 4.5μs for a clock of 50MHz. However, in the presented proposal, some calculations were performed sequentially, that is, for the execution of the forward kinematics it was necessary 150 clock cycles and for the inverse, 225 cycles. The use of partial parallelization in the execution of robotic manipulation algorithms provided a significant increase in system throughput. Nevertheless, it is important to note that there is still room for improvement since all calculations can be computed in parallel.

Another hardware implementation of inverse kinematics was presented in [33]. The device used was a 10-DoF biped robot. A CORDIC implementation was used to perform the trigonometric calculations. The execution time needed to compute the kinematics of the 10 joints in FPGA was of 0.44μs. In this paper, a comparison with a software implementation was also performed, and the time taken to perform the same calculations was 3342μs, i.e., the gain on execution, or speedup, on custom FPGA hardware was 7595×. The resulting error between both implementations was acceptable for this specific control.

In [34], an FPGA implementation of the forward and inverse kinematics of a 5-DoF device was presented. The hardware was developed using a fixed point representation where 32 bits were used for the angles representation and 15 bits for the fractional part. For the device spatial positioning, 16 bits were used of which 7 bits for the fractional part. In the implementation of trigonometric functions, a combination of techniques using lookup tables (LUTs) and Taylor series was used. To perform the necessary calculations, a finite-state machine model (FSM) was used to reduce the use of hardware resources; however, the use of such FSM generated a sequential computation of the robotic manipulation algorithms. In this model, the forward kinematics implementation achieved a runtime of 680ns and the inverse 940ns, that is, for the 50MHz clock, the forward kinematics took 34 clock cycles and the inverse kinematics took 47 cycles. Using such approaches to reduce the use of hardware resources increases computation runtime. For tactile device applications, it is important to optimize the runtime rather than the use of hardware resources.

Similarly, an FPGA implementation of forward and inverse kinematics for a 7-DoF device was presented in [35]; however, only 3-DoF required to control the device movement were implemented in hardware. The proposal used a 32-bit fixed point representation and a CORDIC was used to execute the trigonometric functions. To validate the proposal, the FPGA was set to receive the three reference angles, perform the forward kinematics and then the inverse. The model was developed based on pipeline and the operating frequency used was of 100MHz. As a result, the model calculation took 2μs to perform the entire kinematics algorithm, which represented 200 clock cycles.

In this context, it is possible to realize that the use of FPGA-based computing can accelerate haptic device control algorithms. Unlike traditional hardware that processes information sequentially, FPGA enables parallel information processing. However, most studies from the literature have developed partially parallel implementations, that is, implementations in which parts of the used algorithms are executed sequentially. Unlike the research previously mentioned, this study presents a new approach in which the execution of the robotic manipulation algorithms are performed in a full-parallel hardware implementation. This proposed implementation provides a latency reduction for the tactile devices and enables tactile internet applications.

## 3. Discrete Model of the Tactile Internet

**Figure 1:**
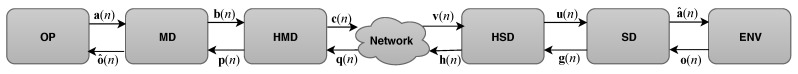
Proposed discrete model of the tactile internet system.

A discrete model of the tactile internet system is proposed and presented in Figure 1. This model consists of seven subsystems: the Operator (OP), Master Device (MD), Hardware of the MD (HMD), Network (NW), Hardware of the SD (HSD), Slave Device (SD) and the Environment (ENV). It is assumed that the signals are sampled at time $t_{s}$.

The OP is an entity responsible for generating stimuli that can be in the form of position signals, speed, force, image, sound or any other. These stimuli are sent to the devices involved so that some kind of task can be performed in some kind of environment. The environment, the ENV subsystem, receives the stimuli from the OP and generates feedback signals associated with sensations such as reactive force information and tactile information that are sent back to the OP. The interaction between the OP and the ENV is performed through the master and slave devices, MD and SD, respectively.

Specifically in this work, MD is characterized as a local device, SD as remote one and both of them are responsible for transforming the stimuli and sensations associated with OP and ENV into signals to be processed. Tactile devices (MD and SD) can take the form of robotic manipulators, haptic devices, tactile gloves and others that may be developed in the future. In the coming years, the introduction of new types of sensors and actuators is expected that will form the basis for the development of new tactile devices.

Although there are no tactile internet standards nor products yet, it can be affirmed that future tactile devices will be integrated with a hardware responsible for all operational metrics and calculations. Within this conjecture, this work adds a couple of modules to the discrete model (as per Figure 1), called HMD and HSD. HMD is responsible for performing all transformations and calculations associated with MD, and HSD performs the equivalent operations for the SD. Several algorithms associated with transformation, compression, control, prediction will be under the responsibility of these two modules. The authors from [36] present a few approaches focusing on the reduction of kinesthetic data and tactile signal compression, which can be applied to the model.

Based on the model presented in Figure 1, the signals generated by the OP can be characterized by the array a(n) expressed as

(1) $$a\left(n\right)=\left[a_{1}\left(n\right),\cdots ,a_{i}\left(n\right),\cdots ,a_{N_{OP}}\left(n\right)\right],$$

where $a_{i}\left(n\right)$ is the *i*-th stimulus at the *n*-th instant and $N_{OP}$ is the total number of stimuli signals generated by the OP. At every *n*-th moment the stimulus array, a(n), is received by the MD which transforms the stimuli into a set of $N_{MD}$ signals expressed as

(2) $$b\left(n\right)=\left[b_{1}\left(n\right),\cdots ,b_{i}\left(n\right),\cdots ,b_{N_{MD}}\left(n\right)\right],$$

where $b_{i}\left(n\right)$ is the *i*-th signal generated by MD at the *n*-th instant. It can be stated that at each *n*-th moment a set of stimuli a(n) generates a set of signals b(n) that depend on the type of MD and the sensor set associated with the device. Especially important is the fact that the signals generated by MD, b(n), have heterogeneous characteristics in which each *i*-th signal $b_{i}\left(n\right)$ can represent an angle, spatial coordinate, pixel of an image, audio sample or any other information associated with a stimulus generated by OP. In practice, the signals grouped by the b(n) array originate from sensors coupled to the MD and the amount of data may vary according to the amount of information to be sent, $N_{MD}$.

The set of signals, expressed by b(n) are sent to the HMD (Figure 1) which has the function of processing this information before sending it to the NW subsystem. Calculations associated with calibration, linear and nonlinear transformations and signal compression are performed by the HMD. Essentially, the majority of the computational effort of MD is in this subsystem. At each *n*-th instant $t_{s}$ the HMD processes the array b(n) generating an information array c(n) expressed by

(3) $$c\left(n\right)=\left[c_{1}\left(n\right),\cdots ,c_{i}\left(n\right),\cdots ,c_{N_{HMD}^{f}}\left(n\right)\right],$$

where $c_{i}\left(n\right)$ is the *i*-th signal generated by HMD towards the subsystem NW at the *n*-th instant $t_{s}$ and $N_{HMD}^{f}$ is the numbers of signals. $N_{HMD}^{f}<N_{MD}$ is expected to minimize latency during the transmission in the NW subsystem.

The NW subsystem, as shown in Figure 1, characterizes the communication medium that links OP to ENV. In this model, the data propagates through two different channels called the forward channel, that transmits the OP data towards the ENV, and the backwards channel, that transmits the ENV signals towards the OP. The signal transmitted by the forward and backwards channels may be disturbed and delayed. In the case of the forward channel, the received signal, v(n), may be expressed as

(4) $$v\left(n\right)=\left[v_{1}\left(n\right),\cdots ,v_{i}\left(n\right),\cdots ,v_{N_{HMD}^{f}}\left(n\right)\right],$$

where

(5) $$v_{i}\left(n\right)=c_{i}\left(n-d_{i}^{f}\left(n\right)\right)+r_{i}^{f}\left(n\right)$$

in which, $r_{i}^{f}\left(n\right)$ represents the added noise and $d_{i}^{f}\left(n\right)$ represents a delays associated with the *i*-th information sent in c(n). In this model, the noise can be characterized as a random Gaussian variable of zero mean and $\sigma_{rf}^{2}$ variance, and the delays are characterized as integers, that is, they occur at a granularity of $t_{s}$. It is important to note that the NW subsystem can take the shape of the Internet, a metropolitan network (MAN), a local area network (LAN), or even a direct connection between an MD and a workstation or computer.

As shown in Figure 1, the HSD receives the v(n) signal through the forward channel and has the role of generating control signals to the SD through the signal

(6) $$u\left(n\right)=\left[u_{1}\left(n\right),\cdots ,u_{i}\left(n\right),\cdots ,u_{N_{HSD}^{f}}\left(n\right)\right],$$

where $N_{HSD}^{f}$ is the number of control signals and $u_{i}\left(n\right)$ is *i*-th control signal at the *n*-th instant $t_{s}$ associated with the array u(n). It is important to note that there may be various types of SD: from real robotic handlers to virtual tools in computational environments. Thus, it can be stated without loss of generality that HSD can perform an inverse processing to HMD in addition to specific algorithms associated with the type of SD. For example, if the SD is a robotic handler, HSD must additionally implement closed loop control algorithms, whereas if SD is a virtual arm HSD must implement positioning algorithms for a given virtual reality platform. SD does not have to correspond directly with MD, e.g., MD can be a glove while SD is a drone. However, it is desirable that the stimulus generated by the SD is a copy of the stimulus generated by the OP, that is, within the model presented in Figure 1, it can be understood that SD generate a signal expressed as

(7) $$\hat{a}\left(n\right)=\left[{\hat{a}}_{1}\left(n\right),\cdots ,{\hat{a}}_{i}\left(n\right),\cdots ,{\hat{a}}_{N_{OP}}\left(n\right)\right],$$

where ${\hat{a}}_{i}\left(n\right)$ is an estimate of the *i*-th stimulus $a_{i}\left(n\right)$ generated by the OP. Thus, the estimate of the stimulus generated by OP, ${\hat{a}}_{i}\left(n\right)$, is applied to the ENV subsystem representing a given real or virtual environment in which OP is interacting.

In the backwards direction, the stimulus actions generated by OP, a(n), and represented by $\hat{a}\left(n\right)$, receives a group of reactions from the ENV subsystem that can be characterized in the model by the set of signals expressed by

(8) $$o\left(n\right)=\left[o_{1}\left(n\right),\cdots ,o_{i}\left(n\right),\cdots ,o_{N_{ENV}}\left(n\right)\right],$$

where $N_{ENV}$ is the number of stimulus signals and $o_{i}\left(n\right)$ is *i*-th stimulus signal at the *n*-th instant $t_{s}$. Reaction signals grouped into o(n) can be in the form of strength, touch, temperature, etc.

Reaction signals are captured by the SD that turns this information into electrical signals from real or virtual sensors, if the SD is in a virtual reality environment. After capturing this information the SD transmits these signals to the HSD. In the model presented in Figure 1, the signals generated by the SD are expressed as

(9) $$g\left(n\right)=\left[g_{1}\left(n\right),\cdots ,g_{i}\left(n\right),\cdots ,g_{N_{SD}}\left(n\right)\right],$$

where $g_{i}\left(n\right)$ is the *i*-th signal generated by the SD at the *n*-th instant of time, $t_{s}$ and $N_{SD}$ is the amount of signals. The HSD in turn processes this information and sends to the NW subsystem through the array h(n), expressed by

(10) $$h\left(n\right)=\left[h_{1}\left(n\right),\cdots ,h_{i}\left(n\right),\cdots ,h_{N_{HSD}^{b}}\left(n\right)\right],$$

where $h_{i}\left(n\right)$ is the *i*-th signal generated by HSD at the *n*-th instant of time, $t_{s}$ and $N_{HSD}^{b}$ is the amount of signals.

The signal received by the HMD through the backwards channel of the NW subsystem can be expressed as

(11) $$q\left(n\right)=\left[q_{1}\left(n\right),\cdots ,q_{i}\left(n\right),\cdots ,q_{N_{HSD}^{b}}\left(n\right)\right],$$

where

(12) $$q_{i}\left(n\right)=h_{i}\left(n-d_{i}^{b}\left(n\right)\right)+r_{i}^{b}\left(n\right)$$

in which, $r_{i}^{b}\left(n\right)$ represents an added noise and $d_{i}^{b}\left(n\right)$ represents a delay associated with the *i*-th information transmitted in q(n) by the backwards channel. Similarly to the forward channel, noise can also be characterized as a random variable Gaussian of zero mean and variance $\sigma_{rb}^{2}$ and delays are characterized as integers with $t_{s}$ granularity. The HMD processes the q(n) signal information and generates a set of control signals that will act on the MD and can be characterized as

(13) $$p\left(n\right)=\left[p_{1}\left(n\right),\cdots ,p_{i}\left(n\right),\cdots ,p_{N_{HMD}^{b}}\left(n\right)\right],$$

where $p_{i}\left(n\right)$ is the *i*-th signal generated by the HMD at the *n*-th instant of time $t_{s}$ and $N_{HMD}^{b}$ is the number of signals. The MD in turn will synthesize the reaction stimuli generated by the environment, i.e., the ENV subsystem. Based on the model, it is possible to characterize these reaction stimuli as a signal expressed by

(14) $$\hat{o}\left(n\right)=\left[{\hat{o}}_{1}\left(n\right),\cdots ,{\hat{o}}_{i}\left(n\right),\cdots ,{\hat{o}}_{N_{ENV}}\left(n\right)\right],$$

where ${\hat{o}}_{i}\left(n\right)$ is an estimate of the *i*-th stimulus $o_{i}\left(n\right)$ generated in the ENV subsystem. Examples of reaction stimuli generated or synthesized by MD are touch, strength and temperature.

In addition to the latency associated with the NW subsystem that characterizes the communication medium between the OP and ENV subsystems, the MD, HMD, HSD, and SD subsystems also add latency to the system. Based on the work presented in [13,14] these components represent 30% of total latency. The latency of the MD and SD subsystems are associated with sensors and actuators that can be mechanical, electrical, electromechanical and other variations. HMD and HSD latencies are associated with the processing time of the algorithms in these devices and depending on the type of hardware and implementation architecture this latency can be considerably reduced.

## 4. PHANToM Omni Device Model (MD & SD)

**Figure 2:**
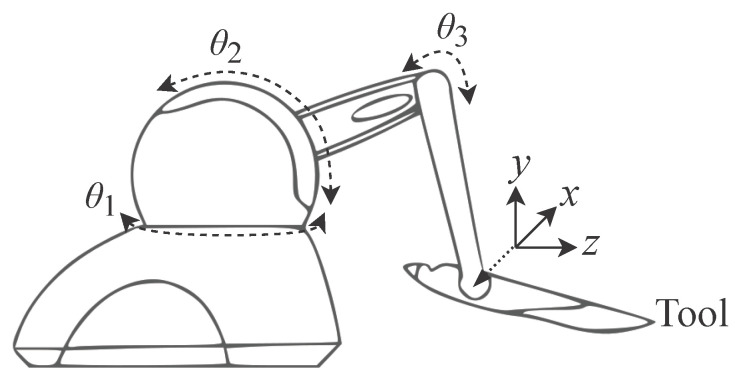
PHANToM Omni—MD and SD.

Based on the scheme presented in Figure 1, this section presents details associated with the MD and SD used as reference for the hardware system proposed in this research. The MD and SD are characterized as a three degree of freedom robotic manipulator, 3-DoF, called the PHANToM Omni [37] (Figure 2). The PHANToM Omni has been widely used in literature as presented in [38,39]. In this work two of this devices are going to be used: one as an MD and the other as a SD.

**Figure 3:**
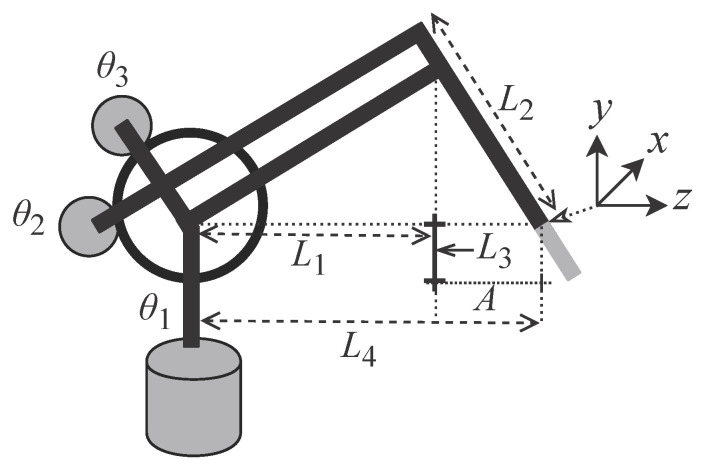
PHANToM Omni structure—MD and SD.

As can be seen from Figure 3, the PHANToM Omni physical structure is formed by a base, an arm with two segments $L_{1}$ and $L_{2}$ which are interconnected by three rotary joints $\theta_{1}$, $\theta_{2}$ and $\theta_{3}$ and a tool. The variables presented in Figure 3 are represented by: $L_{1}$ = 0.135 mm, $L_{2}$ = $L_{1}$, $L_{3}$ = 0.025 mm and $L_{4}=L_{1}+A$ where *A* = 0.035 mm as described in [40]. These detailed features of the device are essential for performing the kinematics and dynamic calculations.

### 4.1. Forward Kinematics

The kinematics of manipulative devices makes use of the relationship between operational coordinates and joint coordinates. Forward kinematics (FK) correlates the angular variables of the joints with the Cartesian system. That is, given an array of joint coordinates it is possible to determine the spatial position of the tool through the equation that can be expressed by

(15) $$x=-\sin\left(\theta_{1}\right)\left(L_{2}\sin\left(\theta_{3}\right)+L_{1}\cos\left(\theta_{2}\right)\right),$$

(16) $$y=-L_{2}\cos\left(\theta_{3}\right)+L_{1}\sin\left(\theta_{2}\right)+L_{3},$$

(17) $$\begin{array}{c} z=L_{2}\cos\left(\theta_{1}\right)\sin\left(\theta_{3}\right)\\ +L_{1}\cos\left(\theta_{1}\right)\cos\left(\theta_{2}\right)-L_{4} \end{array}$$

where *x*, *y* and *z* are variables that determine the spatial position of the tool in the Cartesian plane.

### 4.2. Inverse Kinematics

In inverse kinematics (IK), the relationship between the joint angles and the Cartesian system is reversed, that is, given the spatial position of the tool it may be possible to determine the joint coordinates. The solution to this process is not as straightforward as in the direct kinematics. In direct kinematics, the position of the tool is determined solely by the displacements of the joints. In inverse kinematics, equations are composed of nonlinear calculations formed by trigonometric functions. Depending on the manipulator structure, multiple solutions may be possible for the same tool position, or there may be no solution for a particular set of tool positions. Based on the works [40,41,42], the value of $\theta_{1}$ can be defined through the equation expressed by

(18) $$\theta_{1}=-\mathrm{atan}2\left(x,z+L_{4}\right)$$

where *x* and *z* represent coordinates in the Cartesian plane and $L_{4}$ corresponds to the size of the the arm segments, as shown in Figure 3.

To calculate the other two joints $\theta_{2}$ and $\theta_{3}$ it is necessary to perform intermediate calculations. Thus, one can obtain *R*, *r*, β, γ and α through the equations

(19) $$R=\sqrt{x^{2}+{\left(z+L_{4}\right)}^{2}},$$

(20) $$r=\sqrt{(x^{2}+z+L_{4}{)}^{2}+{\left(y-L_{3}\right)}^{2}},$$

(21) $$\gamma =\mathrm{acos}\left(\frac{L_{1}^{2}-L_{2}^{2}+r^{2}}{2L_{1}r}\right),$$

(22) $$\beta \left(n\right)=\mathrm{atan}2\left(y-L_{3},R\right),$$

and

(23) $$\alpha =\mathrm{acos}\left(\frac{L_{1}^{2}+L_{2}^{2}-r^{2}}{2L_{1}L_{2}}\right).$$

After performing the intermediate calculations it is possible to calculate $\theta_{2}$ through the equation

(24) $$\theta_{2}=\gamma +\beta .$$

Finally, the value corresponding to the $\theta_{3}$ joint can be obtained through the equation

(25) $$\theta_{3}=\theta_{2}+\alpha -\frac{\pi }{2}.$$

### 4.3. Kinesthetic Feedback Force

The kinesthetic feedback force allows the environment to be “felt”, i.e., when the SD comes into physical contact with an object, the MD will receive a counter force. This model can be implemented through the equation

(26) $$\tau =J^{T}F,$$

where τ defines the torque array that will be applied to each joint ($\theta_{1}$, $\theta_{2}$ and $\theta_{3}$) of the PHANToM Omni associated with the MD, $J^{T}$ is the transpose of the Jacobian matrix and F is the force array resulting from the interaction of SD with ENV. The torque array τ can be expressed as

(27) $$\tau =\left[\tau_{1},\tau_{2},\tau_{3}\right].$$

The J Jacobian matrix incorporates structural information about the handler and it is identified as

(28) $$J=\left[\begin{array}{ccc} J_{11} & J_{12} & J_{13}\\ J_{21} & J_{22} & J_{23}\\ J_{31} & J_{32} & J_{33} \end{array}\right],$$

where

(29) $$J_{11}=-\cos\left(\theta_{1}\right)\left(L_{2}\sin\left(\theta_{3}\right)+L_{1}\cos\left(\theta_{2}\right)\right),$$

(30) $$J_{21}=0,$$

(31) $$J_{31}=-L_{1}\cos\left(\theta_{2}\right)\sin\left(\theta_{1}\right)-L_{2}\sin\left(\theta_{3}\right)\sin\left(\theta_{1}\right),$$

(32) $$J_{12}=L_{1}\sin\left(\theta_{1}\right)\sin\left(\theta_{2}\right),$$

(33) $$J_{22}=L_{1}\cos\left(\theta_{2}\right),$$

(34) $$J_{32}=-L_{1}\sin\left(\theta_{2}\right)\cos\left(\theta_{1}\right),$$

(35) $$J_{13}=-L_{2}\sin\left(\theta_{1}\right)\cos\left(\theta_{3}\right),$$

(36) $$J_{23}=L_{2}\sin\left(\theta_{3}\right),$$

and

(37) $$J_{33}=L_{2}\cos\left(\theta_{3}\right)\cos\left(\theta_{1}\right).$$

The force array F is expressed as

(38) $$F=\left[F_{x},F_{y},F_{z}\right]$$

and can be obtained through sensors internal or external to the device. According to Equation (26), the τ torque array representing the resulting force at each joint can be defined as

(39) $$\tau_{1}=J_{11}F_{x}+J_{21}F_{y}+J_{31}F_{z},$$

(40) $$\tau_{2}=J_{12}F_{x}+J_{22}F_{y}+J_{32}F_{z},$$

and

(41) $$\tau_{3}=J_{13}F_{x}+J_{23}F_{y}+J_{33}F_{z}.$$

## 5. Simulated Tactile Internet Model

**Figure 4:**
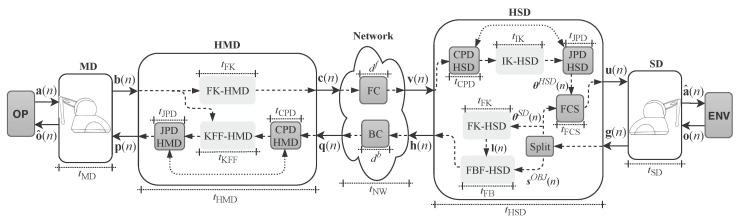
Detailed discrete model of a tactile internet system.

Figure 1 and Figure 4 details the structure used for the hardware design in FPGA, in which a given operator, OP, handles a PHANToM Omni on the master side, MD, which is connected to HMD that, in this case, is a dedicated FPGA hardware. Data are transmitted through the network, the NW subsystem, to HSD which is also a dedicated hardware in FPGA. The HSD is also connected to a PHANToM Omni that interacts with the environment, the ENV subsystem. Figure 4 also details the backwards direction from the ENV and the OP.

The OP is modeled as an information source responsible for generating a spatial trajectory through discrete signals expressed in the a(n) array. At each *n*-th instant $t_{s}$ the OP sends three variables $x^{OP}\left(n\right)$, $y^{OP}\left(n\right)$ and $z^{OP}\left(n\right)$ representing the positioning of the MD tool (Figure 2 and Figure 3) in the Cartesian space an this is expressed by

(42) $$a\left(n\right)=\left[x^{OP}\left(n\right),y^{OP}\left(n\right),z^{OP}\left(n\right)\right].$$

Both devices, master and slave PHANToM Omni, and the structures that form the system were modeled and simulated on Matlab/Simulink [43] and Xilinx System Generator. This step simulates the spatial movement of the MD tool by the operator, that is, at each instant of time, $t_{s}$, a spatial movement is performed and a new signal a(n) is generated by the OP.

The PHANToM Omni has encoders at its three joints that translate spatial positioning at the three angles $\theta_{1}$, $\theta_{2}$ and $\theta_{3}$ (Figure 2 and Figure 3). Thus, based on Figure 4, it can be said that MD converts the signal a(n) into a signal expressed as

(43) $$b\left(n\right)=\left[\theta_{1}^{MD}\left(n\right),\theta_{2}^{MD}\left(n\right),\theta_{3}^{MD}\left(n\right)\right]$$

and forwards it to the HMD at every *n*-th instant of time $t_{s}$.

Then, as can be seen in Figure 4, the b(n) signal propagates to the HMD, which on receiving the signal transforms the joint positioning angles, b(n), into spatial position by calculating the FK according to Equations (15)–(17). All equations are implemented in FPGA through a hardware module called the FK-HMD. The equations are implemented in parallel which can significantly increase the processing time. The use of FK is motivated by an reduction of the amount of information utilized, i.e., for a *N*-DoF robotic manipulator *N* joint angles will be generated and that can be converted into only three values associated with the spatial position of the tool, *x*, *y* and *z*. On the other hand, the use of this strategy increases the amount of calculations to be performed by the MD, which is compensated by the parallel implementation of the algorithm in FPGA. It is essential to note that the use of custom hardware operating in parallel allows processing time not to be substantially affected by *N*.

Based on Section 3, after the FK calculation by the FK-HMD hardware module, a new discrete signal is created that can be expressed by

(44) $$c\left(n\right)=\left[x^{HMD}\left(n\right),y^{HMD}\left(n\right),z^{HMD}\left(n\right)\right]$$

where $x^{HMD}\left(n\right)$, $y^{HMD}\left(n\right)$ and $z^{HMD}\left(n\right)$ are the values of the spatial coordinate array generated by the HMD to be sent to HSD via the communication medium, NW. The FK-HMD hardware module generates a new c(n) array every *n*-th instant of time.

After the transmission through the forward channel, here called FC, the signal received by the HSD can be expressed as

(45) $$v\left(n\right)=\left[x^{HSD}\left(n\right),y^{HSD}\left(n\right),z^{HSD}\left(n\right)\right].$$

Based on Equation (5) the spatial coordinate signal received by HSD can be expressed as

(46) $$x^{HSD}\left(n\right)=x^{HMD}\left(n-d_{x}^{f}\left(n\right)\right)+r_{x}^{f}\left(n\right),$$

(47) $$y^{HSD}\left(n\right)=y^{HMD}\left(n-d_{y}^{f}\left(n\right)\right)+r_{y}^{f}\left(n\right),$$

and

(48) $$z^{HSD}\left(n\right)=z^{HMD}\left(n-d_{z}^{f}\left(n\right)\right)+r_{z}^{f}\left(n\right)$$

where $d_{x}^{f}\left(n\right)$, $d_{y}^{f}\left(n\right)$, $d_{z}^{f}\left(n\right)$, $r_{x}^{f}\left(n\right)$, $r_{y}^{f}\left(n\right)$ and $r_{z}^{f}\left(n\right)$ are the delays and noises associated with CF.

As, in this case, the Slave PHANToM Omni, SD, copies the movement of the master PHANToM Omni, MD, it is necessary for the HSD to perform a feedback control system on the three joints of the PHANToM Omni slave, here expressed as

(49) $$\theta^{SD}\left(n\right)=\left[\theta_{1}^{SD}\left(n\right),\theta_{2}^{SD}\left(n\right),\theta_{3}^{SD}\left(n\right)\right]$$

that is, $\theta_{1}^{SD}\left(n\right)$, $\theta_{2}^{SD}\left(n\right)$, $\theta_{3}^{SD}\left(n\right)$ are control variables associated with DS. The control system illustrated in Figure 4 as FCS shall minimize the error, $e^{FCS}\left(n\right)$, between $\theta^{SD}\left(n\right)$ and the reference signal $\theta^{HSD}\left(n\right)$ characterized as

(50) $$\theta^{HSD}\left(n\right)=\left[\theta_{1}^{HSD}\left(n\right),\theta_{2}^{HSD}\left(n\right),\theta_{3}^{HSD}\left(n\right)\right]$$

where

(51) $$e\left(n\right)=\theta^{HSD}\left(n\right)-\theta^{SD}\left(n\right)and$$

and

(52) $$\left[\begin{array}{c} e_{1}^{FCS}\left(n\right)\\ e_{2}^{FCS}\left(n\right)\\ e_{3}^{FCS}\left(n\right) \end{array}\right]=\left[\begin{array}{c} \theta_{1}^{HSD}\left(n\right)\\ \theta_{2}^{HSD}\left(n\right)\\ \theta_{3}^{HSD}\left(n\right) \end{array}\right]-\left[\begin{array}{c} \theta_{1}^{SD}\left(n\right)\\ \theta_{2}^{SD}\left(n\right)\\ \theta_{3}^{SD}\left(n\right) \end{array}\right].$$

The $\theta^{SD}\left(n\right)$ signal is obtained from the SD via sensors (encoders) at the SD joints and the $\theta^{HSD}\left(n\right)$ signal is obtained from the IK-HSD hardware module shown in Figure 4. This hardware module implements all inverse kinematics equations presented in Section 4.2, i.e., Equations (18)–(25). There are several techniques and approaches that can be used in the FCS module ranging from more traditional techniques such as a proportional-integral-derivative controller [44] to more innovative artificial intelligence based techniques [45,46].

The CPD-HSD and JPD-HSD modules, illustrated in Figure 4, represent the algorithms of prediction and detection in cartesian space and joints, respectively. These modules are responsible for minimizing the latency and noise added by the FC associated with the tactile internet system (Equations (46)–(48)). Depending on the prediction and detection technique used, the HSD may use only one of the modules, namely the CPD-HSD or JPD-HSD. There is still no consensus about whether the Cartesian space or joints is the best for minimizing latency and noise inserted by the channel. There are several works in the literature that present proposals using only one of the spaces and proposals that try to use the information from both simultaneously.

Similarly to the FCS module, approaches ranging from the more traditional techniques up to more innovative techniques based on artificial intelligence have been used in the CPD-HSD and JPD-HSD modules [47,48,49,50,51]. Thus, it can be said that $\theta^{HSD}\left(n\right)$ is an estimate of the b(n) signal generated by the MD.

At each *n*-th time, the FCS acts on the SD through the u(n) signal, detailed in Figure 1 and Figure 4, which in the case of the PHANToM Omni can be expressed as

(53) $$u^{HSD}\left(n\right)=\left[\tau_{1}^{HSD}\left(n\right),\tau_{2}^{HSD}\left(n\right),\tau_{3}^{HSD}\left(n\right)\right]$$

where $\tau_{i}^{HSD}\left(n\right)$ is the *i*-th torque applied every *i*-th joint. The FCS will act as a tracking mechanism, making the SD follow the path traveled by the MD. Finalizing the data stream associated with the forward channel, it can be said that the $\hat{a}\left(n\right)$ signal is formed by an estimate of the spatial position generated by the OP, $\hat{a}\left(n\right)$, i.e.,

(54) $$\hat{a}\left(n\right)=\left[{\hat{x}}^{OP}\left(n\right),{\hat{y}}^{OP}\left(n\right),{\hat{z}}^{OP}\left(n\right)\right].$$

The interaction of the PHANToM Omni, SD, with ENV can vary from free movement to physical contact. When some kind of physical contact occurs, the SD detects the touch and sends this information back to the HSD. As per the model detailed in Figure 4 the ENV sends back to SD the information associated with the contact force in the spatial plane, expressed here as,

(55) $$o\left(n\right)=\left[F_{x}^{ENV}\left(n\right),F_{y}^{ENV}\left(n\right),F_{z}^{ENV}\left(n\right)\right].$$

The value associated with the contact force information can be measured directly through SD-coupled force sensors or indirectly estimated through other types of sensors that may be SD-coupled or inserted into the environment [52]. In the case of the model presented in Figure 4, the SD sends to HSD the objects surface’s spatial positions through sensors spread in the ENV. The signal expressed as

(56) $$s^{OBJ}\left(n\right)=\left[x^{OBJ}\left(n\right),y^{OBJ}\left(n\right),z^{OBJ}\left(n\right)\right]$$

represents the spatial position of the closest object from the SD tool. Thus, based on the information already described, every *n*-th time $t_{s}$ the SD sends to the HSD a signal characterized by the array g(n) expressed as

(57) $$g\left(n\right)=\left[\theta^{SD}\left(n\right),s^{OBJ}\left(n\right)\right].$$

In the HSD, when the signal g(n) is received, the Split module separates the $\theta^{SD}\left(n\right)$ signal and sends it to the FCS and the FK-HSD hardware module. In addition, the signal $s^{OBJ}\left(n\right)$ is sent to the FB-HSD hardware module, as detailed in Figure 4. The FK-HSD hardware module performs the forward kinematics calculation similarly to FK-HMD and thus the current spatial position of the SD tool in the environment, ENV, can be obtained. Every *n*-th instant $t_{s}$ FK-HSD generates a signal expressed as

(58) $$l\left(n\right)=[x^{ENV}\left(n\right),y^{ENV}\left(n\right),z^{ENV}\left(n\right)]$$

where $x^{ENV}\left(n\right)$, $y^{ENV}\left(n\right)$ and $z^{ENV}\left(n\right)$ are the spatial position of the tool in the ENV module from $\theta^{SD}\left(n\right)$. The FBF-HSD hardware module implements the calculations associated with the generation of the feedback force from the contact between the tool and the object. Based on the work presented in [52] the contact force, represented by the h(n) signal, can be expressed as

(59) $$h\left(n\right)=\left[F_{x}^{HSD}\left(n\right),F_{y}^{HSD}\left(n\right),F_{z}^{HSD}\left(n\right)\right],$$

where

(60) $$F_{x}^{HSD}\left(n\right)=h_{x}\left(n\right)\left(x^{OBJ}\left(n\right)-x^{ENV}\left(n\right)\right),$$

(61) $$F_{y}^{HSD}\left(n\right)=h_{y}\left(n\right)\left(y^{OBJ}\left(n\right)-y^{ENV}\left(n\right)\right),$$

and

(62) $$F_{z}^{HSD}\left(n\right)=h_{z}\left(n\right)\left(z^{OBJ}\left(n\right)-z^{ENV}\left(n\right)\right).$$

In these equations, the constants $h_{x}\left(n\right)$, $h_{y}\left(n\right)$ and $h_{z}\left(n\right)$ represent the elasticity coefficients associated with the object. It is important to note that in this model the h(n) signal is a synthesized version of the real force value here characterized by the o(n) array.

After the feedback force calculation process, as illustrated in Figure 4, the h(n) signal is transmitted to the HMD via the backwards channel (BC) which, similarly to FC, adds latency and noise. The signal received by the HMD can be expressed as

(63) $$q\left(n\right)=[F_{x}^{HMD}\left(n\right),F_{y}^{HMD}\left(n\right),F_{z}^{HMD}\left(n\right)]$$

where

(64) $$F_{x}^{HMD}\left(n\right)=F_{x}^{HSD}\left(n-d_{x}^{b}\left(n\right)\right)+r_{x}^{b}\left(n\right),$$

(65) $$F_{y}^{HMD}\left(n\right)=F_{y}^{HSD}\left(n-d_{y}^{b}\left(n\right)\right)+r_{y}^{b}\left(n\right),$$

and

(66) $$F_{z}^{HMD}\left(n\right)=F_{z}^{HSD}\left(n-d_{z}^{b}\left(n\right)\right)+r_{z}^{b}\left(n\right)$$

where $d_{x}^{b}\left(n\right)$, $d_{y}^{b}\left(n\right)$, $d_{z}^{b}\left(n\right)$, $r_{x}^{b}\left(n\right)$, $r_{y}^{b}\left(n\right)$ and $r_{z}^{b}\left(n\right)$ are the latencies and the noises associated with the BC.

Similarly to HSD, the HMD will minimize the effect of latency and noise from operations of Cartesian and joint space. For HMD, the calculations associated with the Cartesian space will be performed by the CPD-HMD module and associated with the joint space by the JPD-HMD module. In addition to the prediction and detection calculations, the HMD must transform the force signals received through signal q(n) into a torque to be applied to the MD joints which is accomplished by the KFF-HMD hardware module. KFF-HMD implements the Equations (39)–(41) presented in Section 4.3 and generate the signal expressed as

(67) $$p\left(n\right)=\left[\tau_{1}^{HMD}\left(n\right),\tau_{2}^{HMD}\left(n\right),\tau_{3}^{HMD}\left(n\right)\right]$$

where $\tau_{i}^{HMD}\left(n\right)$ is the torque associated with the *i*-th joint of the MD. Since the PHANToM Omni is a haptic device, it already has a built-in control system, FCS, which uses as reference signal the torques associated with the p(n) array.

After applying the torques to the MD joints via the p(n) signal, the OP receives the feedback force signal, in other words, it feels the object touched by the SD in the ENV. This sensation is identified in by the $\hat{o}\left(n\right)$ signal expressed as

(68) $$\hat{o}\left(n\right)=\left[{\hat{F}}_{x}^{ENV}\left(n\right),{\hat{F}}_{y}^{ENV}\left(n\right),{\hat{F}}_{z}^{ENV}\left(n\right)\right].$$

As illustrated in Figure 4, the MD, HMD, NW, HSD, and SD subsystems have the following runtimes: $t_{MD}$, $t_{HMD}$, $t_{NW}$, $t_{HSD}$ and $t_{SD}$, respectively. The sum of these, times taking into account the forward direction (between OP and ENV) and the backwards direction (between ENV and OP), represent the total system latency that can be expressed as

(69) $$t_{\mathrm{latency}}=2\left(t_{\mathrm{MD}}+t_{\mathrm{HMD}}+t_{\mathrm{NW}}+t_{\mathrm{HSD}}+t_{\mathrm{SD}}\right).$$

Some works presented in the literature review agree that the ideal requirement is that $t_{\mathrm{latency}}\leq 1\;\mathrm{ms}$, on the other hand, other works point out that the latency requirement can be expresses as $t_{\mathrm{latency}}\leq 10\;\mathrm{ms}$, depending on the application [9,10,11,12,53]. Considering that 30% of the total latency time $t_{\mathrm{latency}}$ is spent by MD, HMD, HSD, and SD, it can be understood that

(70) $$\left(t_{\mathrm{MD}}+t_{\mathrm{HMD}}+t_{\mathrm{HSD}}+t_{\mathrm{SD}}\right)\leq \frac{0.3t_{\mathrm{latency}}}{2}.$$

Assuming an equal time division among MD, HMD, HSD, and SD it is possible to affirm that the time associated with hardware, $t_{\mathrm{hardware}}$, whether the master, HMD, or the slave device, HSD, can be expressed as

(71) $$t_{\mathrm{HMD}}=t_{\mathrm{HSD}}=t_{\mathrm{hardware}}\leq \frac{0.3t_{\mathrm{latency}}}{8}.$$

Taking the 1ms constraints into consideration and substituting this value in Equation (71), it is possible to affirm that the hardware time, $t_{\mathrm{hardware}}$, must meet the $t_{\mathrm{hardware}}\leq 37.5\;\mu s$ constraint for all cases (condition 1ms) or the $t_{\mathrm{hardware}}\leq 375\;\mu s$ constraint for some specific cases (10ms condition).

Recent studies from the literature show that the 1ms restriction ($t_{\mathrm{hardware}}\leq 37.5\;\mu s$) is difficult to achieve using hardware devices based on embedded systems such as microprocessors and microcontrollers [26,54]. The 10ms restriction ($t_{\mathrm{hardware}}\leq 375\;\mu s$) is achieved in specific cases where SD is a virtual environment and HSD is a high performance processor computer [53]. Thus this work aims to minimize the execution time in HMD, $t_{\mathrm{HMD}}$, and HSD, $t_{\mathrm{HSD}}$, using FPGA. In other words, the target is to achieve a $t_{\mathrm{hardware}}\leq 37.5\;\mu s$.

This paper presents a hardware reference model for the FK-HMD, KFF-HMD, IK-HSD, FK-HSD, and FBF-HSD modules illustrated in Figure 4. The complete model that will be presented in detail in the next section makes use of a parallel implementation methodology in which high throughput is prioritized, i.e., the execution time of the modules $t_{\mathrm{FK}}$, $t_{\mathrm{KFF}}$, $t_{\mathrm{IK}}$ and $t_{\mathrm{FBF}}$, illustrated in Figure 4.

This work does not propose dedicated hardware reference models for the CPD-HSD, JPD-HSD, CPD-HMD, JPD-HMD and FCS modules as there are several techniques and algorithms that can be applied to them. However, considering the hardware time constraints, $t_{\mathrm{hardware}}$, it is noted that it is also important to use dedicated hardware structures with as FPGA-based circuits for these modules. Studies in the literature foresee the use of AI based techniques for these modules; however, it is essential to note that AI techniques and algorithms implemented on general purpose processor-based hardware platforms can lead to higher processing times [19,20,21,22,23,24,25].

## 6. Implementation Description

**Figure 5:**
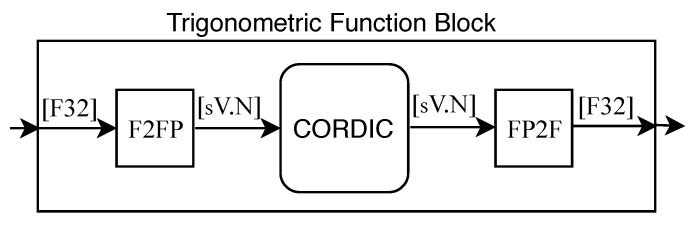
Proposed circuit for calculating trigonometric functions—TFB.

The FK-HMD and KFF-HMD hardware modules associated with the master device (HMD) and the IK-HSD, FK-HSD, and FBF-HSD hardware modules associated with the slave device (HSD) (Figure 4) were designed using a parallel implementation in order to prioritize the processing speed. The implementations were designed in FPGA using a hybrid scheme with fixed point and floating point representation in distinct parts of the proposed architecture. In the portions that adopt the fixed point format, the variables follow a notation expressed as [sV.N] indicating that the variable is formed by *V* bits of which *N* bits are intended for the fractional part and the *s* symbol indicates that the variable is signed. In this case, the number of bits intended for the integer part is V−N−1. For the representation of floating point variables, the notation [F32] is adopted. Most of the implemented circuits were designed using a 32-bit single precision (IEEE754) floating point format representation. The fixed point format was used only on the circuit that implements the trigonometric function block (TFB) module, as illustrated in Figure 5. TFB is the module responsible for performing trigonometric operations through the hardware implementation of CORDIC (COordinate Rotation DIgital Computer) [55]. For that, a Xilinx CORDIC IP Core was used. This implementation uses data representation in a fixed-point format using the [s16.13] representation.

As illustrated in Figure 5, the TFB module receives data from external circuits in the 32-bits floating point standard. A conversion to the fixed point numeric representation type represented by the [s16.13] notation is performed through the Float to Fixed-point (F2FP) module that has been implemented in hardware. After the CORDIC hardware operations are performed, the data in the fixed point format is transformed back to the 32-bit floating point through the Fixed-point to Float (FP2F) module which was also implemented in hardware.

Several of the proposed methods to be presented use the constants $L_{1}$, $L_{2}$, $L_{3}$ and $L_{4}$. They represent physical characteristics of the PHANToM Omni device as illustrated in Figure 2. These constants use the 32-bit floating point numeric representation.

### 6.1. Forward Kinematics (FK-HMD and FK-HSD)

As illustrated in Figure 4, both the hardware associated with the master device (HMD) and the hardware associated with the slave device (HSD) implement forward kinematics through the FK-HMD and FK-HSD modules, respectively. These modules have the same FPGA-implemented circuit, differing only in the input and output signals. They are designed to work with three input signals, one for each component of the angular positioning of the device’s joints, and three output signals, one for each component of the the positioning of the device’s tool in the Cartesian system. The input signals are $\theta_{1}\left[F32\right]\left(n\right)$, $\theta_{2}\left[F32\right]\left(n\right)$ and $\theta_{3}\left[F32\right]\left(n\right)$ and the output signals are x[F32](n), y[F32](n) and z[F32](n). For FK-HMD, the input signals represent the $\theta_{1}^{MD}\left[F32\right]\left(n\right)$, $\theta_{2}^{MD}\left[F32\right]\left(n\right)$ and $\theta_{3}^{MD}\left[F32\right]\left(n\right)$ signals, and the output signals represent the $x^{HMD}\left[F32\right]\left(n\right)$, $y^{HMD}\left[F32\right]\left(n\right)$ and $z^{HMD}\left[F32\right]\left(n\right)$ signals. In the case of the FK-HSD module, the input signals represent the signals $\theta_{1}^{SD}\left[F32\right]\left(n\right)$, $\theta_{2}^{SD}\left[F32\right]\left(n\right)$ and $\theta_{3}^{SD}\left[F32\right]\left(n\right)$ and the output signals represent the signals $x^{ENV}\left(n\right)$, $y^{ENV}\left(n\right)$ and $z^{ENV}\left(n\right)$. At every *n*-th instant all the computation performed in order to calculate the forward kinematics are executed in parallel.

**Figure 6:**
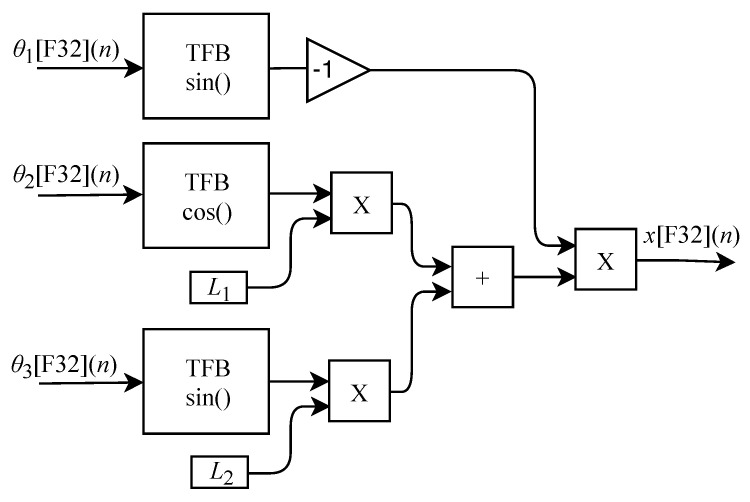
Proposed forward kinematics circuit for obtaining the x[F32](n) spatial coordinate (Equation (15))—FK-HMD and FK-HSD.

Based on Equation (15), the algorithm used for calculating x[F32](n) was implemented in FPGA through the generic circuit illustrated in Figure 6. The circuit was designed to work with three input signals $\theta_{1}\left[F32\right]\left(n\right)$, $\theta_{2}\left[F32\right]\left(n\right)$ and $\theta_{3}\left[F32\right]\left(n\right)$ and one output signal. These signals are forwarded to TFB sub circuits where sine and cosine calculations are performed. For this process the constants $L_{1}$ and $L_{2}$, three multipliers, one inverter and one adder are used.

**Figure 7:**
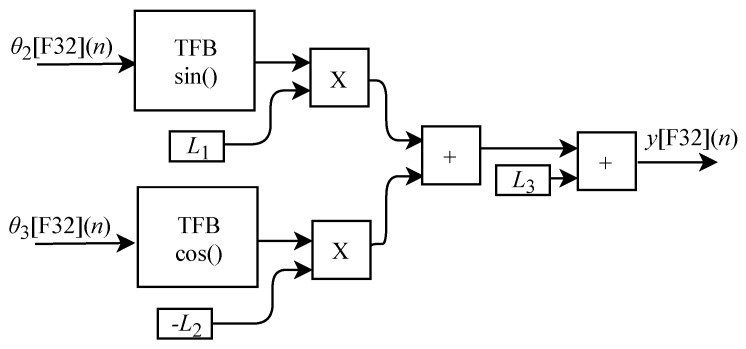
Proposed forward kinematics circuit for obtaining the y[F32](n) spatial coordinate (Equation (16))—FK-HMD and FK-HSD.

The calculation of y[F32](n) based on Equation (16) was implemented in FPGA through the generic circuit shown in Figure 7. The circuit was designed to work with two input signals $\theta_{2}\left[F32\right]\left(n\right)$ and $\theta_{3}\left[F32\right]\left(n\right)$ and one output signal. These signals are routed to TFB sub circuits to perform sine and cosine calculations. In the process flow two multipliers, two adders, one inverter and the constants $L_{1}$ and $L_{2}$ are used.

**Figure 8:**
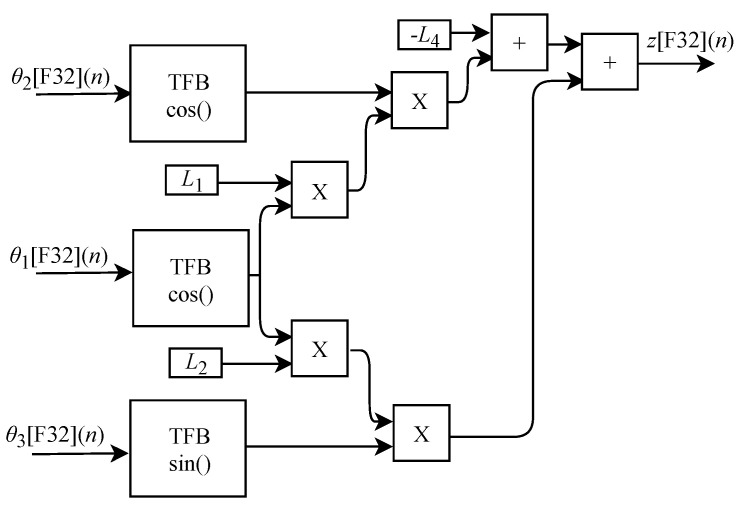
Proposed forward kinematics circuit for obtaining the z[F32](n) spatial coordinate (Equation (17))—FK-HMD and FK-HSD.

The generic circuit illustrated in Figure 8 was implemented in FPGA to perform the calculation of z[F32](n) and it is based on Equation (17). The circuit is designed to work with three input signals $\theta_{1}\left[F32\right]\left(n\right)$, $\theta_{2}\left[F32\right]\left(n\right)$ and $\theta_{3}\left[F32\right]\left(n\right)$ and one output signal. These signals are routed to TFB sub circuits in order to perform sine and cosine calculations. In the process flow four multipliers, two adders, one inverter and the constants $L_{1}$, $L_{2}$ and $L_{4}$ are used.

In the FK-HMD module the $\theta_{1}^{MD}\left[F32\right]\left(n\right)$, $\theta_{2}^{MD}\left[F32\right]\left(n\right)$ and $\theta_{3}^{MD}\left[F32\right]\left(n\right)$ input signals are received through the b(n) array (Equation (43) in Section 5), then all calculation are performed in parallel resulting in the c(n) array (Equation (44) in Section 5) with the $x^{HMD}\left[F32\right]\left(n\right)$, $y^{HMD}\left[F32\right]\left(n\right)$ and $z^{HMD}\left[F32\right]\left(n\right)$ signals as shown in Figure 4. For the FK-HSD module the $\theta_{1}^{SD}\left[F32\right]\left(n\right)$, $\theta_{2}^{SD}\left[F32\right]\left(n\right)$ and $\theta_{3}^{SD}\left[F32\right]\left(n\right)$ input signals enter the module via the $\theta^{SD}\left(n\right)$ array (Equation (49) in Section 5) and after performing all parallel computations, the resulting signals $x^{ENV}\left(n\right)$, $y^{ENV}\left(n\right)$ and $z^{ENV}\left(n\right)$ are output from the module via the l(n) array (Equation (49) in Section 5).

### 6.2. Inverse Kinematics (IK-HSD)

The hardware associated with the slave device (HSD) implements the inverse kinematics through the IK-HSD module, as shown in Figure 4. The IK-HSD FPGA-implemented circuit is designed to work with three input signals $x^{HSD}\left[F32\right]\left(n\right)$, $y^{HSD}\left[F32\right]\left(n\right)$ and $z^{HSD}\left[F32\right]\left(n\right)$ and three output signals $\theta_{1}^{HSD}\left[F32\right]\left(n\right)$, $\theta_{2}^{HSD}\left[F32\right]\left(n\right)$ and $\theta_{3}^{HSD}\left[F32\right]\left(n\right)$. However, to calculate $\theta_{2}^{HSD}\left[F32\right]\left(n\right)$ (Equation (24)) and $\theta_{3}^{HSD}\left[F32\right]\left(n\right)$ (Equation (25)) it is first necessary to perform intermediate calculations to obtain the values of R[F32](n), r[F32](n), β[F32](n), γ[F32](n) and α[F32](n)

**Figure 9:**
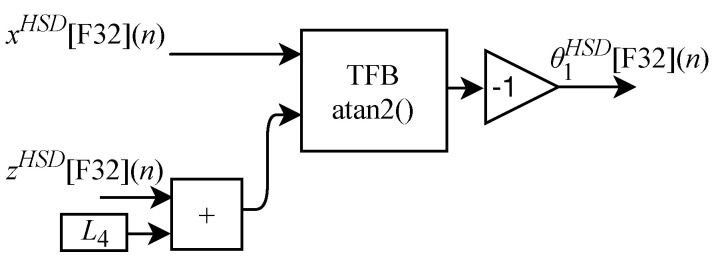
Proposed inverse kinematics circuit for obtaining the $\theta_{1}^{HSD}\left[F32\right]\left(n\right)$ angular position (Equation (18))—IK-HSD.

**Figure 10:**
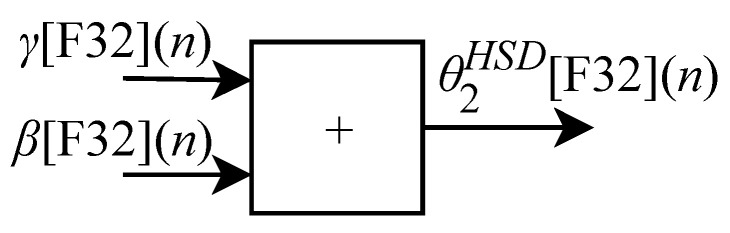
Proposed inverse kinematics circuit for obtaining the $\theta_{2}^{HSD}\left[F32\right]\left(n\right)$ angular position (Equation (24))—IK-HSD.

**Figure 11:**
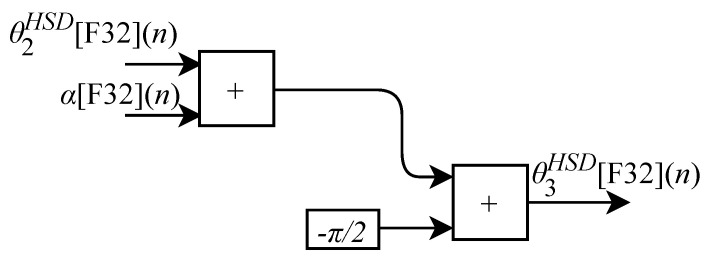
Proposed inverse kinematics circuit for obtaining the $\theta_{3}^{HSD}\left[F32\right]\left(n\right)$ angular position (Equation (25))—IK-HSD.

Based on Equations (18), (24) and (25), algorithms for calculating $\theta_{1}^{HSD}\left[F32\right]\left(n\right)$, $\theta_{2}^{HSD}\left[F32\right]\left(n\right)$ and $\theta_{3}^{HSD}\left[F32\right]\left(n\right)$ were implemented in FPGA through the generic circuits illustrated in Figure 9, Figure 10 and Figure 11 respectively.

**Figure 12:**
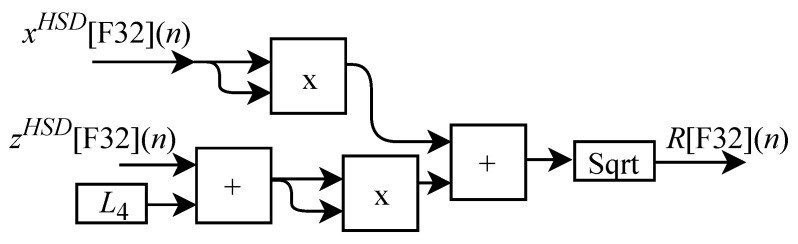
Proposed circuit to perform the calculation of R[F32](n) (Equation (19))—IK-HSD.

**Figure 13:**
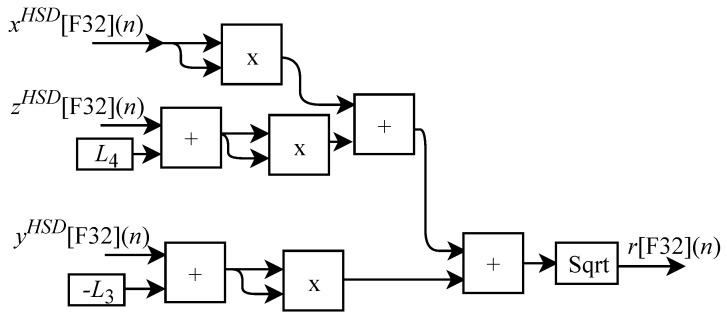
Proposed circuit to perform the calculation of r[F32](n) (Equation (20))—IK-HSD.

As already described, and according to the illustrations shown in Figure 10 and Figure 11, to perform the calculations of $\theta_{2}^{HSD}\left[F32\right]\left(n\right)$ and $\theta_{3}^{HSD}\left[F32\right]\left(n\right)$ it is first necessary to perform the intermediate calculations of γ[F32](n) (Equation (21)), β[F32](n) (Equation (22)) and α[F32](n) (Equation (23)). However, these calculations depend on the calculation of R[F32](n) and r[F32](n). Then, when the IK-HSD module receives the input signals at every *n*-th instant the circuit shown in Figure 9 performs the calculation of $\theta_{1}^{HSD}\left[F32\right]\left(n\right)$ in parallel with the generic circuits illustrated in Figure 12 and Figure 13 which were implemented in FPGA to perform the calculation of R[F32](n) and r[F32](n) based on Equations (19) and (20).

The circuit shown in Figure 12 used to obtain R[F32](n), is designed to work with two input signals $x^{HSD}\left[F32\right]\left(n\right)$ and $z^{HSD}\left[F32\right]\left(n\right)$ and one output signal. This design contains two multipliers, two adders, the $L_{4}$ constant and a sub-circuit called Sqrt, which was implemented in hardware to calculate the square root.

The r[F32](n) calculation is performed through the circuit shown in Figure 13. This circuit is designed to work with three input signals $x^{HSD}\left[F32\right]\left(n\right)$, $y^{HSD}\left[F32\right]\left(n\right)$ and $z^{HSD}\left[F32\right]\left(n\right)$ and one output signal. The circuit consists of three multipliers, four adders, one inverter, the constants $L_{3}$ and $L_{4}$, and, again, the Sqrt sub-circuit.

**Figure 14:**
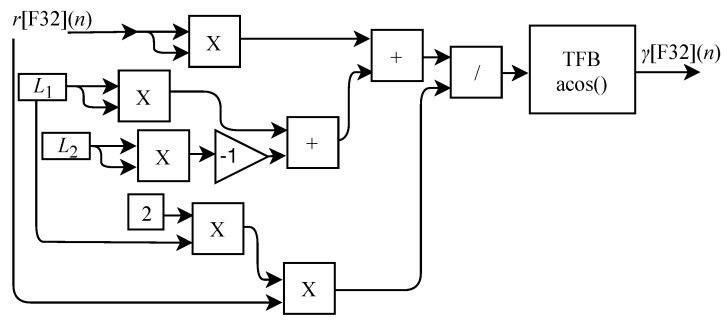
Proposed circuit to perform the calculation of γ[F32](n) (Equation (21))—IK-HSD.

**Figure 15:**
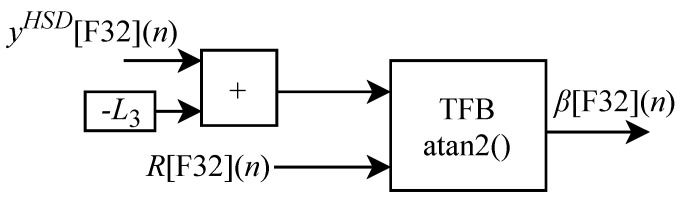
Proposed circuit to perform the calculation of β[F32](n) (Equation (22))—IK-HSD.

**Figure 16:**
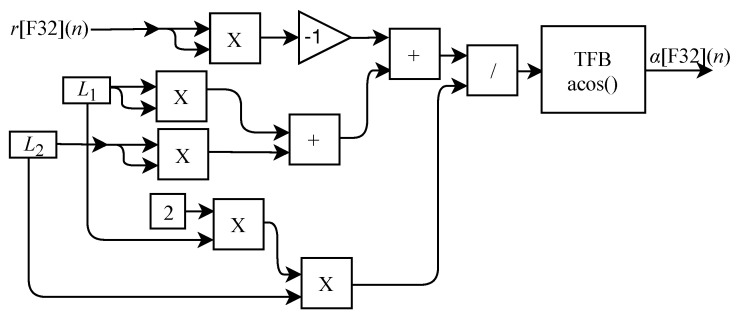
Proposed circuit to perform the calculation of α[F32](n) (Equation (23))—IK-HSD.

After the parallel processing of $\theta_{1}^{HSD}\left[F32\right]\left(n\right)$, R[F32](n) and r[F32](n), the circuits responsible for calculating γ[F32](n), β[F32](n) and α[F32](n) are also executed in parallel through the FPGA implementations of the generic circuits illustrated in Figure 14, Figure 15 and Figure 16. The value of γ[F32](n) is obtained through the circuit shown in Figure 14 which is based on Equation (21). The circuit is designed to work with an input signal r[F32](n) and one output signal. It consists of five multipliers, two adder, one divisor, one TFB sub-circuit to calculate the arccosine and the constants $L_{1}$ and $L_{2}$.

The circuit for obtaining β[F32](n) illustrated in Figure 15 is based on Equation (22) and is designed to work with two input signals $y^{HSD}\left[F32\right]\left(n\right)$ and R[F32](n) and one output signal. The circuit is composed of one adder, one inverter, a TFB sub-circuit to perform the arctangent calculation and the $L_{3}$ constant.

The value of α[F32](n) is obtained from the circuit shown in Figure 16 which is based on Equation (23) and is designed to work with an input signal r[F32](n) and one output signal. The circuit is composed of five multipliers, two adders, one inverter, one divider, one TFB sub-circuit to perform the arccosine calculation and the constants $L_{1}$ and $L_{2}$.

To complete the process, after performing the calculations of β[F32](n), γ[F32](n) and α[F32](n), it is possible to obtain the $\theta_{2}^{HSD}\left[F32\right]\left(n\right)$ and $\theta_{3}^{HSD}\left[F32\right]\left(n\right)$ values in parallel through the circuits shown in Figure 10 and Figure 11.

### 6.3. KKinesthetic Feedback Force (KFF-HMD)

**Figure 17:**
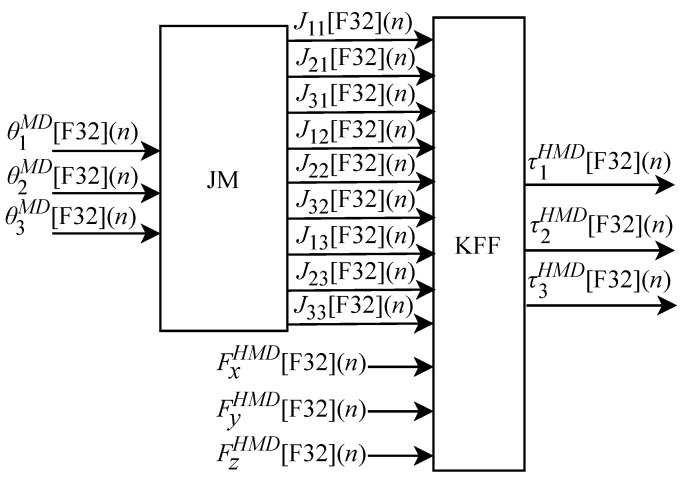
Proposed circuit to calculate kinesthetic feedback force (Equation (26))—KFF-HMD.

As illustrated in Figure 4, the hardware associated with the master device (HMD) implements the kinesthetic feedback force through the KFF-HMD module. Based on Equation (26), the KFF-HMD module was implemented in FPGA through the generic circuit illustrated in Figure 17. This circuit is composed of sub-circuits that correspond to parts of Equation (26). The sub-circuit called JM, described in Equation (28), is responsible for calculating the Jacobian matrix. The KFF sub-circuit makes the relationship between the Jacobian matrix (JM) module and the force array from Equation (38).

The circuit shown in Figure 17 has the input signals $\theta_{1}^{MD}\left[F32\right]\left(n\right)$, $\theta_{2}^{MD}\left[F32\right]\left(n\right)$ and $\theta_{3}^{MD}\left[F32\right]\left(n\right)$ that are received from the master device (MD) and also the $F_{x}\left[F32\right]\left(n\right)$, $F_{y}\left[F32\right]\left(n\right)$ and $F_{z}\left[F32\right]\left(n\right)$ signals that are received from the hardware associated to the slave device (HSD). The three output signals are: $\tau_{1}^{HMD}\left[F32\right]\left(n\right)$, $\tau_{2}^{HMD}\left[F32\right]\left(n\right)$ and $\tau_{3}^{HMD}\left[F32\right]\left(n\right)$.

**Figure 18:**
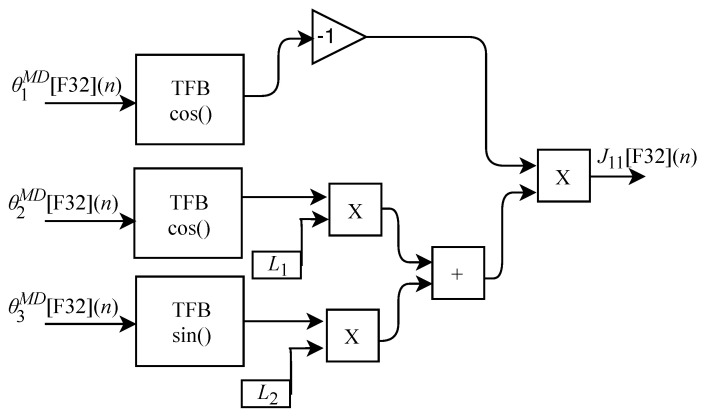
Proposed circuit to calculate the Jacobian matrix $J_{11}\left[F32\right]\left(n\right)$ (Equation (29))—JM.

The JM module that represents the sub-circuit responsible for performing the Jacobian matrix calculation consists of nine elements: $J_{11}\left[F32\right]\left(n\right)$, $J_{21}\left[F32\right]\left(n\right)$, $J_{31}\left[F32\right]\left(n\right)$, $J_{12}\left[F32\right]\left(n\right)$, $J_{22}\left[F32\right]\left(n\right)$, $J_{32}\left[F32\right]\left(n\right)$, $J_{13}\left[F32\right]\left(n\right)$, $J_{23}\left[F32\right]\left(n\right)$ and $J_{33}\left[F32\right]\left(n\right)$. The calculation of $J_{21}\left[F32\right]\left(n\right)$ based on Equation (30) does not have an associated circuit since its value is 0, i.e., $J_{21}\left[F32\right]\left(n\right)=0$. Based on Equation (29), the algorithm for calculating $J_{11}\left[F32\right]\left(n\right)$ was implemented in FPGA according to the generic circuit illustrated in Figure 18. The circuit was designed to work with three input signals and one output signal. It uses the constants $L_{1}$ and $L_{2}$ and has three TFB sub-circuits: two for performing the cosine calculation and one for obtaining the sine value.

**Figure 19:**
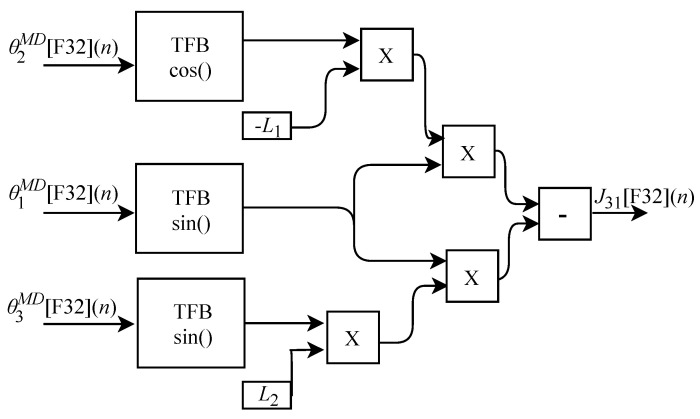
Proposed circuit to calculate the Jacobian matrix $J_{31}\left[F32\right]\left(n\right)$ (Equation (31))—JM.

The calculation of $J_{31}\left[F32\right]\left(n\right)$, based on Equation (31), was implemented in FPGA according to the generic circuit illustrated in Figure 19. The circuit was designed to work with three input signals and one output signal. The circuit has three TFB modules, two for sine calculation and one for cosine value and uses the $L_{1}$ and $L_{2}$ constants.

**Figure 20:**
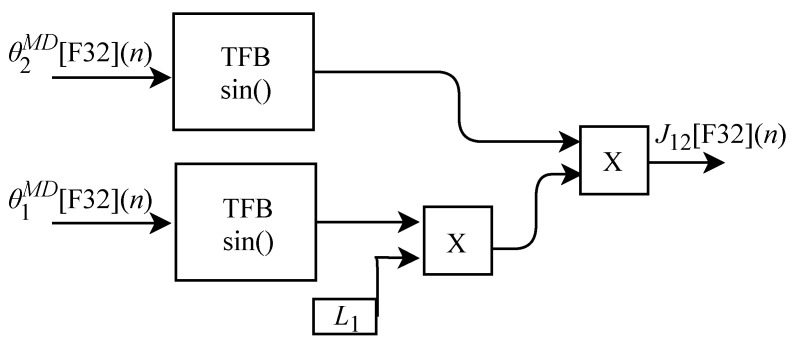
Proposed circuit to calculate the Jacobian matrix $J_{12}\left[F32\right]\left(n\right)$ (Equation (32))—JM.

The generic circuit illustrated in Figure 20 was implemented in FPGA to perform the calculation of $J_{12}\left[F32\right]\left(n\right)$ and is based on Equation (32). The circuit was designed to work with two input signals and one output signal. The circuit has two TFB sub circuits to perform sine calculation and uses the $L_{1}$ constant.

**Figure 21:**
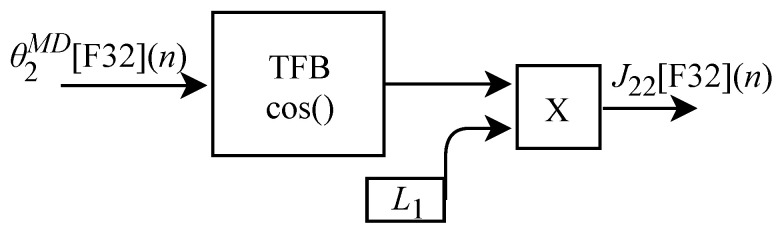
Proposed circuit to calculate the Jacobian matrix $J_{22}\left[F32\right]\left(n\right)$ (Equation (33))—JM.

Based on Equation (33), the algorithm for calculating $J_{22}\left[F32\right]\left(n\right)$ was implemented in FPGA according to the generic circuit illustrated in Figure 21. The circuit was designed to work with one input signal and one output signal. The circuit has a TFB sub-circuit to perform cosine calculation and uses the constant $L_{1}$.

**Figure 22:**
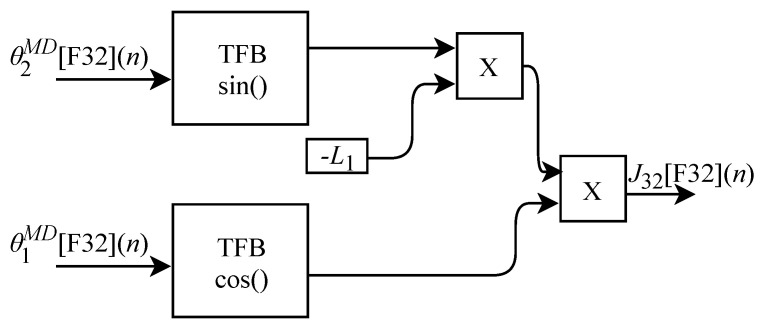
Proposed circuit to calculate the Jacobian matrix $J_{32}\left[F32\right]\left(n\right)$ (Equation (34))—JM.

The calculation of $J_{32}\left[F32\right]\left(n\right)$ based on Equation (34) was implemented in FPGA according to the generic circuit illustrated in Figure 22. The circuit was designed to work with two input signals and one output signal. In addition to the use of the constant $L_{1}$, the circuit has two TFB sub circuits, one for performing the cosine calculation and one for the sine.

**Figure 23:**
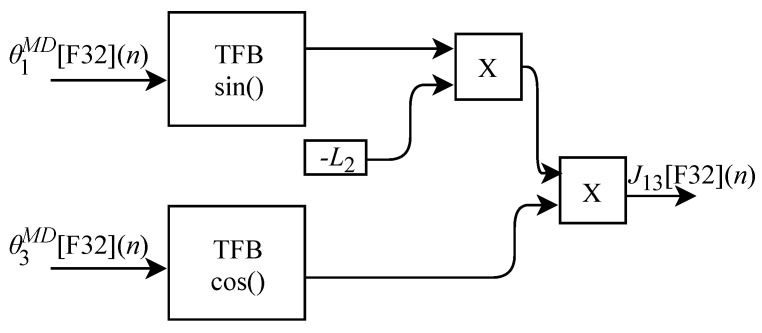
Proposed circuit to calculate the Jacobian matrix $J_{13}\left[F32\right]\left(n\right)$ (Equation (35))—JM.

The generic circuit illustrated in Figure 23 was implemented in FPGA to perform the calculation of $J_{13}\left[F32\right]\left(n\right)$ and which is based on Equation (35). The circuit was designed to work with two inputs and one output signal. In addition to using the constant $L_{2}$, the circuit has two TFB sub circuits, one for performing cosine calculation and one for the sine.

**Figure 24:**
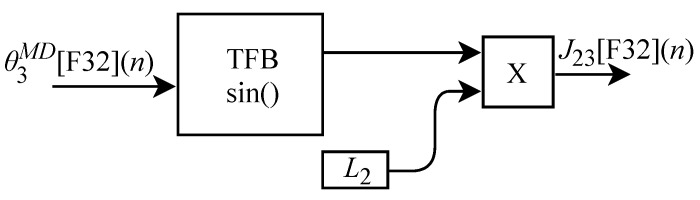
Proposed circuit to calculate the Jacobian matrix $J_{23}\left[F32\right]\left(n\right)$ (Equation (36))—JM.

Based on Equation (36), the algorithm for calculating $J_{23}\left[F32\right]\left(n\right)$ was implemented in FPGA according to the generic circuit illustrated in Figure 24. The circuit was designed to work with one input signal and one output signal. The circuit contains a TFB sub-circuit to perform the sine calculation and uses the $L_{2}$ constant.

**Figure 25:**
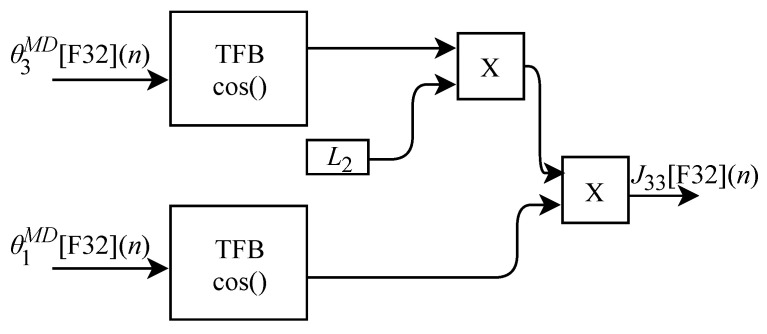
Proposed circuit to calculate the Jacobian matrix $J_{33}\left[F32\right]\left(n\right)$ (Equation (37))—JM.

The calculation of $J_{33}\left[F32\right]\left(n\right)$, based on Equation (37), was implemented in FPGA according to the generic circuit illustrated in Figure 25. The circuit was designed to work with two input signals and one output signal. In addition to the use of constant $L_{2}$, the circuit has two TFB sub-circuits to perform the cosine calculation.

All displayed circuits related to the JM sub-circuits are calculated in parallel at each *n*-th instant. The results are then sent to the KFF module which also performs the calculations of $\tau_{1}^{HMD}\left[F32\right]\left(n\right)$, $\tau_{2}^{HMD}\left[F32\right]\left(n\right)$ and $\tau_{3}^{HMD}\left[F32\right]\left(n\right)$ in parallel. The KF circuit shown in Figure 17 is designed to work with twelve input signals and three output signals.

**Figure 26:**
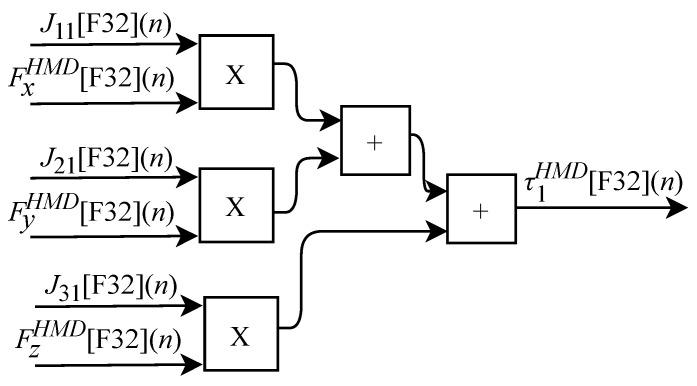
Proposed circuit to calculate the torque of the $\tau_{1}^{HMD}\left[F32\right]\left(n\right)$ joint (Equation (39))—KFF.

Based on Equation (39), the algorithm for calculating $\tau_{1}^{HMD}\left[F32\right]\left(n\right)$ was implemented in FPGA according to the generic circuit illustrated in Figure 26. The circuit was designed to work with six inputs and one output.

**Figure 27:**
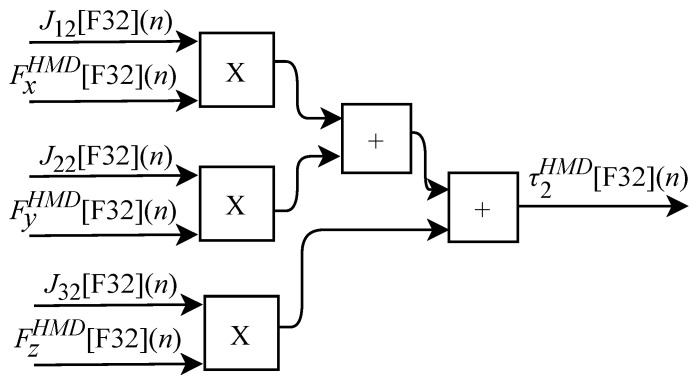
Proposed circuit to calculate the torque of the $\tau_{2}^{HMD}\left[F32\right]\left(n\right)$ joint (Equation (40))—KFF.

The calculation of $\tau_{2}^{HMD}\left[F32\right]\left(n\right)$ based on Equation (40) was implemented in FPGA according to the generic circuit illustrated in Figure 27. The circuit was designed to work with six inputs and one output.

**Figure 28:**
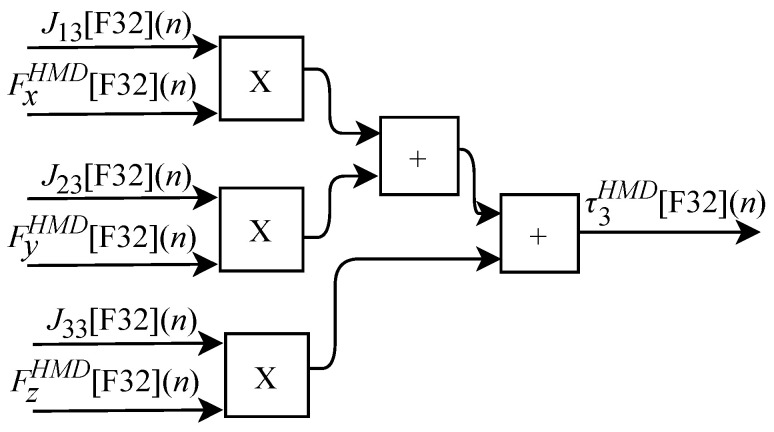
Proposed circuit to calculate the torque of the $\tau_{3}^{HMD}\left[F32\right]\left(n\right)$ joint (Equation (41))—KFF.

The generic circuit illustrated in Figure 28 has been implemented in FPGA to perform the calculation of $\tau_{3}^{HMD}\left[F32\right]\left(n\right)$ and it is based on Equation (41). The circuit was designed to work with six inputs and one output.

### 6.4. Feedback Force (FBF-HSD)

As illustrated in Figure 4 the hardware associated with the slave device (HSD) implements the feedback force via the FBF-HSD module. The FPGA-implemented circuit of the FBF-HSD module is designed to work with six input signals and three output signals. Among the six input variables, $x^{OBJ}\left[F32\right]\left(n\right)$, $y^{OBJ}\left[F32\right]\left(n\right)$ and $z^{OBJ}\left[F32\right]\left(n\right)$ represent the spatial position of the closest object to the SD tool and the other three $x^{ENV}\left[F32\right]\left(n\right)$, $y^{ENV}\left[F32\right]\left(n\right)$ and $z^{ENV}\left[F32\right]\left(n\right)$ represent the spatial position of the SD tool in the ENV module. The three outputs $F_{x}^{HSD}\left[F32\right]\left(n\right)$, $F_{y}^{HSD}\left[F32\right]\left(n\right)$ and $F_{z}^{HSD}\left[F32\right]\left(n\right)$ represent the touch of the tool on the object. The variables $h_{x}$, $h_{y}$ and $h_{z}$ represent the elasticity coefficients associated with the object. All FBF-HSD module calculations are performed in parallel.

**Figure 29:**
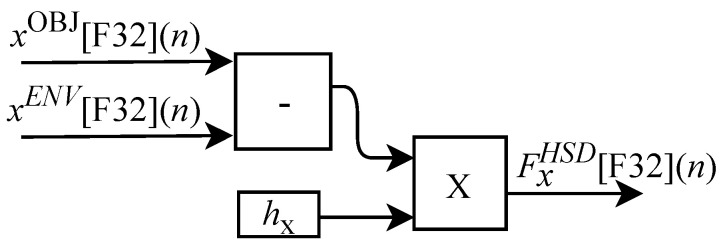
Proposed circuit to calculate the feedback force $F_{x}^{HSD}\left[F32\right]\left(n\right)$ (Equation (60))—FBF-HSD.

Based on Equation (60), the algorithm used for calculating $F_{x}^{HSD}\left[F32\right]\left(n\right)$ was implemented in FPGA according to the generic circuit illustrated in Figure 29. The circuit was designed to work with two inputs signals $x^{OBJ}\left[F32\right]\left(n\right)$ and $x^{ENV}\left[F32\right]\left(n\right)$ and one variable $h_{x}$.

**Figure 30:**
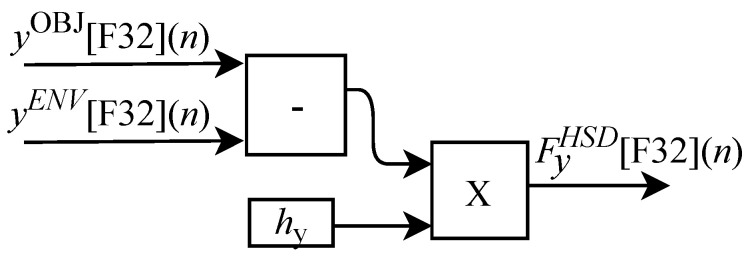
Proposed circuit to calculate the feedback force $F_{y}^{HSD}\left[F32\right]\left(n\right)$ (Equation (61))—FBF-HSD.

The calculation of $F_{y}^{HSD}\left[F32\right]\left(n\right)$, based on Equation (61), was implemented in FPGA according to the generic circuit illustrated in Figure 30. The circuit was designed to work with two input signals $y^{OBJ}\left[F32\right]\left(n\right)$ and $y^{ENV}\left[F32\right]\left(n\right)$ and one variable $h_{y}$.

**Figure 31:**
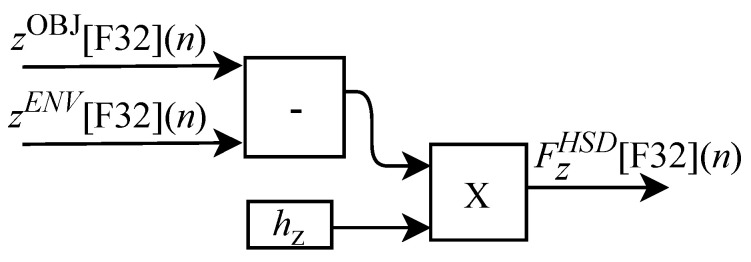
Proposed circuit to calculate the feedback force $F_{z}^{HSD}\left[F32\right]\left(n\right)$ (Equation (62))—FBF-HSD.

The generic circuit shown in Figure 31 was implemented in FPGA to perform the calculation of $F_{z}^{HSD}\left[F32\right]\left(n\right)$ and it is based on Equation (62). The circuit was designed to work with two input signals $z^{OBJ}\left[F32\right]\left(n\right)$ and $z^{ENV}\left[F32\right]\left(n\right)$ and one variable $h_{z}$.

## 7. Results

The entire tactile internet model infrastructure presented in Figure 4 was implemented with the purpose of validating the FPGA hardware implementation. A spatial trajectory that represents the data sent by the OP through the a(n) (Equation (42)) signal was created to validate the entire developed environment.

**Figure 32:**
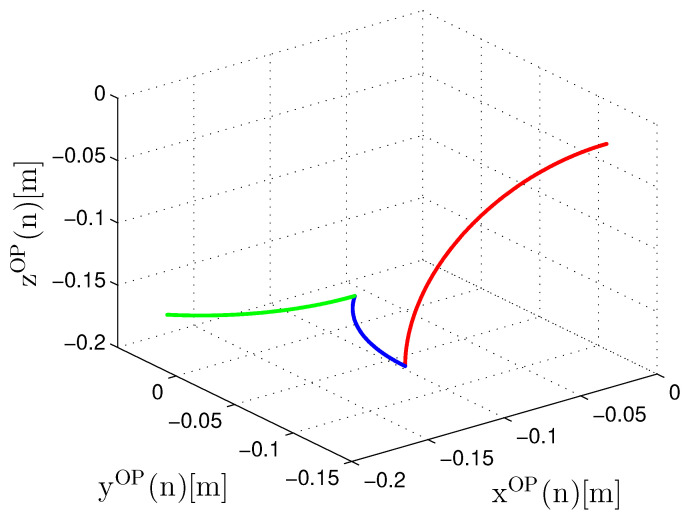
Trajectory used to validate hardware modules.

The created trajectory performs a variation in all of the three angles of the MD articulation (Figure 3). For this, it was first considered that the MD is in the initial angular position expressed as $\theta_{1}^{MD}\left(0\right)=0$, $\theta_{2}^{MD}\left(0\right)=0$ and $\theta_{3}^{MD}\left(0\right)=0$, which corresponds to the spatial position $x^{OP}\left(0\right)=0$, $y^{OP}\left(0\right)=-0.107$ and $z^{OP}\left(0\right)=-0.035$ of the tool as illustrated in Figure 32. Initially, the first joint is moved to $\theta_{1}^{MD}\left(vn\right)=pi/2$ where *v* represents a quantity of samples that is equal to 4 s, thus resulting in the position $x^{OP}\left(vn\right)=-0.132$, $y^{OP}\left(vn\right)=-0.107$ and $z^{OP}\left(vn\right)=-0.167$. Then, the second joint is moved to $\theta_{2}^{MD}\left(vn\right)=pi/4$ which results in the position $x^{OP}\left(vn\right)=-0.093$, $y^{OP}\left(vn\right)=-0.013$ and $z^{OP}\left(vn\right)=-0.167$ and, finally, the third joint moves up to $\theta_{3}^{MD}\left(vn\right)=pi/4$, thus resulting in the $x^{OP}\left(vn\right)=-0.186$, $y^{OP}\left(vn\right)=0.025$ and $z^{OP}\left(vn\right)=-0.167$ position. The path created is within the limits of the device workspace and takes a total time of $t_{1}=12$ s of which 4 s are used to perform the movement of each joint.

In an effort to validate the circuits from the implemented modules in FPGA, equivalent software models were used to compare the results of both implementations. The software models use a 32-bit floating point format while the hardware modules run a parallel implementation with a hybrid representation which uses both a 32-bit floating point and a fixed point representation in different parts of the proposed architecture, as presented in Section 6. In all scenarios, the signal sampling rate (or throughput) was $R_{s}=\frac{1}{t_{s}}$ (samples per second), where $t_{s}$ is the time between the *n*-th samples.

From the experimental results, the mean square error (MSE) between the software model and the hardware implementation proposed by this work was calculated using the MSE which can be expressed as

(72) $$MSE=\frac{1}{Q}\sum_{n=0}^{Q-1}{\left(M^{SW}\left[F32\right]\left(n\right)-M\left[F32\right]\left(n\right)\right)}^{2},$$

where *Q* represents the number of tested samples, $M^{SW}\left[F32\right]\left(n\right)$ corresponds to the variables of the software model and M[F32](n) corresponds to the variables of the model implemented in FPGA.

The quantity of tested samples for the results presented here is Q=1200, which correspond to the quantity of samples of the generated trajectory. The variables that correspond to the hardware model M[F32](n) vary according to the module in which it was implemented. In the case of forward kinematics, as the FK-HMD and FK-HSD modules have the same implementation, the values corresponding to the variables x[F32](n), y[F32](n) and z[F32](n) change according to the respective module. For the FK-HMD module, these variables correspond to $x^{HMD}\left[F32\right]\left(n\right)$, $y^{HMD}\left[F32\right]\left(n\right)$ and $z^{HMD}\left[F32\right]\left(n\right)$ and for the FK-HSD module the same variables correspond to $x^{ENV}\left[F32\right]\left(n\right)$, $y^{ENV}\left[F32\right]\left(n\right)$ and $z^{ENV}\left[F32\right]\left(n\right)$ as presented in Section 6. For inverse kinematics, the variables M[F32](n) of the IK-HSD module correspond to $\theta_{1}^{HSD}\left[F32\right]\left(n\right)$, $\theta_{2}^{HSD}\left[F32\right]\left(n\right)$ and $\theta_{3}^{HSD}\left[F32\right]\left(n\right)$. For the kinesthetic feedback force, the variables M[F32](n) of the KFF-HMD module correspond to $\tau_{1}^{HMD}\left[F32\right]\left(n\right)$, $\tau_{2}^{HMD}\left[F32\right]\left(n\right)$ and $\tau_{3}^{HMD}\left[F32\right]\left(n\right)$. For the feedback force, the variables M[F32](n) of the FBF-HSD module correspond to $F_{x}^{HSD}\left[F32\right]\left(n\right)$, $F_{y}^{HSD}\left[F32\right]\left(n\right)$ and $F_{z}^{HSD}\left[F32\right]\left(n\right)$. Finally, in the MSE equation the $M^{SW}\left[F32\right]\left(n\right)$ corresponds to the same variables as the software-implemented model.

Table 1 shows the mean square error between the software models and the hardware ones proposed in this paper. The obtained MSE-related results prove to be noteworthy, showing that the forward kinematics (FK-HMD and FK-HSD), inverse kinematics (IK-HSD), kinesthetic feedback force (KFF-HMD) and feedback force (FBF-HSD) modules had an acceptable response, even when using a hybrid representation, compared to the software model that uses a floating point representation. It can be observed that for the variables of the FK-HMD and FK-HSD modules the error was in the range of $10^{-08}$, for the IK-HSD module the error was of $10^{-06}$, for the variables of the KFF-HMD module the error was of $10^{-07}$ and for the FBF-HSD module the error was in the range of $10^{-16}$. These values demonstrate that the FPGA implementations presented an equivalent behavior to the software models.

In a hardware implementation, it is important to analyze some requirements post-synthesis such as available hardware usage and the execution time. In the case of FPGAs, the resources are measured through the use of lookup tables (LUTs), Registers and Digital Signal Processors (DSPs) units, to name a few. After validating the hardware-implemented models, synthesis results were obtained using the implementation designed for an FPGA Xilinx Virtex 6 XC6VLX240T-1FF1156. The used Virtex 6 FPGA has 37,680 slices that group 301,440 flip-flops, 150,720 logical cells that can be used to implement logical functions or memories, and 768 DSP cells with multipliers and accumulators. The implementations and results used the Matlab/Simulink and the Xilinx System Generator.

Table 2 presents the post-synthesis results related to FPGA resource utilization, sampling rate, and throughput for the modules FK-HMD, KFF-HMD, FK-HSD, IK-HSD, and FBF-HSD. The first column shows the name of the module, the next three columns called registers, LUTs and multipliers represent the amounts of resources used in the FPGA. The column register represents the number of flip-flops that were used, followed by the total percentage used. The column LUTs represents the number of LUTs that were used, followed by the total percentage used. In addition, the column multipliers represents the number of DPS48 internal multipliers that were used, followed by the total percentage used. The $t_{s}$ column represents the sampling rate in nanoseconds that was obtained for each hardware module. Finally, the $R_{s}$ column displays throughput ($R_{s}=\frac{1}{t_{s}}$) values in mega-samples per second for the hardware modules.

The synthesis results presented in Table 2 show that the resources used for the FK-HMD and FK-HSD modules were the same. This means that each module, individually, used a percentage of 1.01% which is equivalent to 3041 of the available hardware resources for the registers, was used 5.31% with LUTs, and 1.43% for embedded multipliers DSP48. The IK-HSD module had a hardware percentage consumption of 1.04% for registers, 9.36% for LUTs and 3.52% for multipliers. The KFF-HMD module had a consumption of 1.03%, 8.13% and 6.25% for registers, LUTs and multipliers, respectively. Finally, the FBF-HSD module used a percentage of 0.11% for registers, 0.82% for LUTs and 1.17% for multipliers.

Based on data presented in Table 2, the HMD modules (FK-HMD and KFF-HMD) that is associated with the MD device has consumed 6154 (2.04%) for register, 20,259 (13.44%) for LUTs and 59 (7.68%) for multipliers. In the case of hardware associated with the SD device, the HSD modules (FK-HSD, IK-HSD and FBF-HSD) had consumed 6513 (2.16%) for register, 23,351 (15.49%) for LUTs and 47 (6.12%) for multipliers.

The hardware resources consumed by the HMD hardware modules and the HSD hardware modules were very low. Even if all modules are implemented in single hardware, the consumption remains low. The total sum of hardware resources used in the FPGA by all modules (FK-HMD, KFF-HMD, FK-HSD, IK-HSD and FBF-HSD) was: 12,667 (4.20%) for register, 43,610 (28.93%) for LUTs and 106 (13.80%) for multipliers. The low hardware resources consumption demonstrates that the proposed implementations take up little hardware space in the FPGA which allows other separate implementations to be used concomitantly.

As per Table 2, the throughput values, $R_{s}$, obtained were significant. Values of 21.27MSps for the FK-HMD and FK-HSD modules, 4.58MSps for the IK-HSD module, 14.28MSps for the KFF-HMD module and 47.61MSps for the FBF-HSD module were achieved. These results enable critical applications that demand strict time constraints, as is the case with tactile internet applications. The times presented in Table 2 show the critical path (path in the entire design with the maximum delay) on FPGA.

In Table 3, it is possible to see the speedup obtained about latency time constraints. The first column shows the latency constraints for 1ms and 10ms [9,10,11,12]. The second column shows the minimum latency values required for the application to function normally (these values are the estimates obtained by Equation (71) for both time constraints). The third column shows the latency related to the hardware implementation presented here. The speedup, fourth column, is calculated using the values of minimum time, Latency Limit, for each constraint and the time of the proposed hardware. It is worth mentioning that this is an estimate to guide the calculations.

The 1ms restriction corresponds to the maximum latency limit of 37.5μs for acceptable hardware performance. For the 10ms constraint, the maximum limit is 375μs. The value $t_{\mathrm{hardware}}$ that is presented in Table 3 and according to Equation (71), corresponds to the sum of the latencies of the five implemented modules (Table 2), two modules are associated with the MD device (FK-HMD and KFF-HMD) and three modules are associated with the SD device (FK-HSD, IK-HSD, and FBF-HSD).

Thus, the presented value of 403ns in Table 3 corresponds to the sum of the two modules related to the master component, which has a total of 117ns of which 47ns come from the FK-HMD module and 70ns from the KFF-HMD module together with the sum of the three modules referring to the slave component, which has a total of 286ns of which 47ns derives from the FK-HSD module, 218ns from IK-HSD and 21ns from the FBS-HSD module. So for the 1ms constraint, the implementation presented a 93× speedup relative to the 37.5μs, and for the 10ms constraint, the speedup was 930× relatives to the 375μs limit.

The sample rates resulted from the five modules that were implemented in this work were notably fast. The values obtained contributed to the hardware meeting the time constraint limits required in a tactile internet environment. Hardware latency showed values significantly below the required constraints, as shown in Table 3. These values are well below the 30% presented in the literature and due to the fact that the communication medium demands 70% of application latency, this value can be increased as the latency of hardware devices showed to be significantly low. In other words, it can be said that the remaining latency not spent on the hardware devices can be consumed in the network.

It is important to remember that in a more complex tactile internet environment, there are several others more algorithms to be implemented in hardware such as prediction algorithms, dynamic control, AI based techniques, etc. However, as the proposed implementations present low hardware resource consumption, other necessary modules, as the ones previously mentioned, could also be implemented in the same shared hardware since resources would still be available.

Table 4 presents comparisons of the results obtained by the proposed implementation of this work with equivalent results found in works from the state of the art. The first column indicates the references of related works. The next two columns show the used FPGA platform and the amount of degrees of freedom of the used device. The fourth column presents the type of numerical representation used in the implementation and, finally, the last four columns present the times obtained by each reference for latency added by the forward kinematics (FK), inverse kinematics (IK), the kinesthetic force feedback (KFF) and feedback force (FBF) modules, respectively.

As described in Table 4, a hardware model for calculating the forward kinematics of a 5-DoF device is presented in [30]. For the trigonometric calculations, a Taylor series expansion was implemented in FPGA for computing the sine, cosine, and tangent arc functions. The proposed hardware was implemented using a 32-bit floating-point representation. The total time to perform the calculations was 1240ns. The calculations are performed in parallel. Comparing to the forward kinematics (FK) implementation using 32-bit floating-point proposed by this work, the speedup was 26.38× over the model presented in [30].

The work presented in [31] shows the results of an implementation of the inverse kinematics module using floating-point 32-bit representation. Three types of implementations are presented, but only the one with the best performance was compared. For that, it was used an Altera Cyclone IV FPGA, in which a microprocessor system based on the Nios II soft–processor was build. This processor enables to perform operations such as hardware summation multiplication, subtraction division and square root. The equations allow partial parallelization of individual operations, decreasing the computation time. The kinematic model was designed to work with a 3-DoF device, and the time required to calculate is 143000ns. When compared with the proposal of inverse kinematics (IK) presented in this work, which uses 32-bit floating-point representation, this implementation presented a speedup of 655.96× over in relation to the model proposed by [31].

The kinematics models presented in [32] described in Table 4, presented data regarding the forward and inverse kinematics implementations for controlling a 6-DoF device using the 32-bit fixed-point representation. The modules were implemented using 21-bit for the fractional part and 11-bit in the integer part. For the forward kinematics (FK), 3000ns are required to perform all calculations, and for inverse kinematics (IK), 4500ns is required. Based on the results of the implementations presented in this section, the implementation proposed for this work using floating-point representation had a speedup of 63.82× for forward kinematics and 20.64× for the inverse kinematics.

The research presented in [33] proposed a hardware implementation of inverse kinematics to control a 10-DoF device. Although the robotic model has 10 Dof, the equations for the calculations are just composed of subtraction and division calculations. Regarding trigonometric calculations, only the tangent arc is used in the equations, and the square root used through the CORDIC module. The hardware was projected using the 32-bit fixed-point representation, however the amount of bits used in the fractional part was not specified. The architecture proposed to calculate the inverse kinematics requires 440ns to perform the computation. All calculations are performed by the hardware in parallel. Comparing to the inverse kinematics (IK) implementation using 32-bit floating-point proposed by this work, the speedup was 2.01× over the model presented in [33]. The processing time has a lower value when considering the DoFs, but this is due to the fact that the algorithms are less complex.

The authors in [34] present the results of fixed-point implementation for forward and inverse kinematics to control a 5-DoF device, as described in Table 4. The proposed hardware implementation uses the numerical representation of 32-bit (15-bit to fractional part) and 16-bit (7-bit to fractional part) in different parts of the modules. The equations associated with the calculation of the forward and inverse kinematics make use of the trigonometric functions sine, cosine, arctangent, and arccosine. To perform the arctangent and arccosine, the Taylor series expansion was used. The time required to perform the calculations is 680ns and 940ns for forward and inverse kinematics, respectively. Comparing to the floating-point implementation proposed by this work, the speedup was 14.46× for forward kinematic and 4.31× for inverse kinematic over the model presented in [34].

Differently from previous works (Table 4), in [35], the authors present unique hardware for calculating forward and inverse kinematics together. In the proposed model, the 32-bit fixed-point representation was used. The total time to perform the calculation is 2000ns. The time obtained was calculated taking into account the entire process duration; however, separate times for each module were not specified. Given this scenario, by adding the $t_{s}$ FK module time that calculates forward kinematics with the IK module, the total time resulting from both implementations reaches 265ns, according to Table 4. Hence, the hardware presented in the work here developed achieved a 7.54× speedup over the model presented in [35].

Differently from previous works (Table 4), in [35], the authors present unique hardware for calculating forward and inverse kinematics together. The hardware computes all calculations in parallel. The computation of forward and inverse kinematics share the same processing time. An ARM processor was used to make the communication part between the modules, and the FPGA is used to calculate the kinematics model. The CORDIC module was used to perform trigonometric calculations. In the proposed model, the 32-bit fixed-point representation was used. The total time to perform the calculation is 2000ns. The time obtained was calculated taking into account the entire process duration; however, separate times for each module were not specified. Given this scenario, by adding the $t_{s}$ FK module time that calculates forward kinematics with the IK module, the total time resulting from both implementations reaches 265ns, according to Table 4. Hence, the hardware presented in the work here developed achieved a 7.54× speedup over the model presented in [35].

It can be seen from Table 4, that none of the works from the state-of-the-art presented the hardware implementation of all four robotics algorithms that were presented here. It is also noted that just two works used the floating-point numerical representation. The floating-point implementation of robotics algorithms proposed by this work showed significant gains when compared to the works presented in the literature as shown in Table 4. The different amounts of degrees of freedom (DoF) used in the devices can somehow influence in values of sample rate and throughput. Another factor that can also influence these values is in relation to the type of FPGA that is used to perform the synthesis. Due to the fact that the implementation of this work was designed in a parallel architecture, the increase in the amount of DoF does not necessarily reflect in a significant increase in sample rate.

## 8. Conclusions

This paper presented an FPGA hardware reference model for four modules implementing robotics-associated algorithms. The FK-HMD and FK-HSD modules implement the forward kinematics, the IK-HSD module implements the inverse kinematics, the KFF-HMD module implements the kinesthetic feedback force, and the FBF-HSM module implements the feedback force. The parallel FPGA implementation of the four modules is intended to increase the tactile system’s processing speed to meet the latency constraints required for tactile internet applications. The modules were designed using a full-parallel implementation which works on a hybrid scheme that uses fixed point and floating point representation in distinct parts of the architecture. Compared to the state-of-the-art, this work describes and implements four different robotics algorithms in FPGA. The implementations presented in this work achieve higher module processing speed when compared to equivalent implementations from the state-of-the-art. All the modules presented here were analyzed based on the synthesis results, which included the FPGA resource utilization, sampling rate, and yield. Based on the synthesis results, it was observed that the implementations achieved high module processing speed, far below the latency limit of 1ms. Hardware modules accelerated 93× compared to the 37.5μs time constraint. This work demonstrates that using embedded systems on devices such as FPGAs enables parallel algorithm implementation, thus speeding up data processing and minimizing execution time. Runtime gains can make processing time possible for critical applications that require short time constraints or a large amount of data to be processed in a short time frame.

## Figures and Tables

**Table 1:** Mean squared error (MSE) results for floating-point implementation.

Module	Variable	MSE
FK-HMD	$x^{HMD}\left[F32\right]\left(n\right)$	$2.333\times 10^{-8}$
	$y^{HMD}\left[F32\right]\left(n\right)$	$8.316\times 10^{-9}$
	$z^{HMD}\left[F32\right]\left(n\right)$	$1.656\times 10^{-8}$
KFF-HMD	$\tau_{1}^{HMD}\left[F32\right]\left(n\right)$	$1.467\times 10^{-7}$
	$\tau_{2}^{HMD}\left[F32\right]\left(n\right)$	$5.207\times 10^{-9}$
	$\tau_{3}^{HMD}\left[F32\right]\left(n\right)$	$3.350\times 10^{-7}$
FK-HSD	$x^{ENV}\left[F32\right]\left(n\right)$	$2.333\times 10^{-8}$
	$y^{ENV}\left[F32\right]\left(n\right)$	$8.316\times 10^{-9}$
	$z^{ENV}\left[F32\right]\left(n\right)$	$1.656\times 10^{-8}$
IK-HSD	$\theta_{1}^{HSD}\left[F32\right]\left(n\right)$	$3.731\times 10^{-6}$
	$\theta_{2}^{HSD}\left[F32\right]\left(n\right)$	$2.847\times 10^{-6}$
	$\theta_{3}^{HSD}\left[F32\right]\left(n\right)$	$2.702\times 10^{-6}$
FBF-HSD	$F_{x}^{HSD}\left[F32\right]\left(n\right)$	$2.437\times 10^{-16}$
	$F_{y}^{HSD}\left[F32\right]\left(n\right)$	$1.731\times 10^{-16}$
	$F_{z}^{HSD}\left[F32\right]\left(n\right)$	$3.360\times 10^{-16}$

**Table 2:** FPGA resource utilization, sampling rate and throughput results for floating-point format.

Module Name	Registers (Flip-Flops)	LUTs	Multipliers (DSP48)	$t_{s}$ (ns)	$R_{s}$ (MSps)
FK-HMD	3041 (1.01%)	8008 (5.31%)	11 (1.43%)	47	21.27
KFF-HMD	3113 (1.03%)	12,251 (8.13%)	48 (6.25%)	70	14.28
FK-HSD	3041 (1.01%)	8008 (5.31%)	11 (1.43%)	47	21.27
IK-HSD	3149 (1.04%)	14,107 (9.36%)	27 (3.52%)	218	4.58
FBF-HSD	323 (0.11%)	1236 (0.82%)	9 (1.17%)	21	47.61

**Table 3:** Hardware speedup related to the time limits for the 1ms and 10ms latency constraints.

Time Restriction	Latency Limit	$t_{\mathrm{hardware}}$	Speedup
1ms	37.5μs	403ns	93×
10ms	375μs	403ns	930×

**Table 4:** Comparative table with state of the art works.

Reference	Device	DoF	Data Type	FK	IK	KFF	FBF
This work	Virtex 6	3	Floating P.	47ns	218ns	70ns	21ns
[30]	Virtex 2	5	Floating P.	1240ns	-	-	-
[31]	Cyclone IV	3	Floating P.	-	143,000ns	-	-
[32]	Unknown	6	Fixed P.	3000ns	4500ns	-	-
[33]	Cyclone IV	10	Fixed P.	-	440ns	-	-
[34]	Cyclone IV	5	Fixed P.	680ns	940ns	-	-
[35]	Artix 7	3	Fixed P.	2000ns		-	-
